# Rationally designed transition metal hydroxide nanosheet arrays on graphene for artificial CO_2_ reduction

**DOI:** 10.1038/s41467-020-18944-1

**Published:** 2020-10-14

**Authors:** Kang-Qiang Lu, Yue-Hua Li, Fan Zhang, Ming-Yu Qi, Xue Chen, Zi-Rong Tang, Yoichi M. A. Yamada, Masakazu Anpo, Marco Conte, Yi-Jun Xu

**Affiliations:** 1 College of Chemistry, New Campus, Fuzhou University, 350116 Fuzhou, P. R. China; 2 State Key Laboratory of Photocatalysis on Energy and Environment, College of Chemistry, Fuzhou University, 350116 Fuzhou, P. R. China; 3 RIKEN Center for Sustainable Resource Science, Hirosawa, Wako, Saitama, 351-0198 Japan; 4 Department of Applied Chemistry, Graduate School of Engineering, Osaka Prefecture University, Osaka, 599-8531 Japan; 5 Department of Chemistry, University of Sheffield, Sheffield, S3 7HF UK

**Keywords:** Solid-state chemistry, Photocatalysis, Photocatalysis, Graphene

## Abstract

The performance of transition metal hydroxides, as cocatalysts for CO_2_ photoreduction, is significantly limited by their inherent weaknesses of poor conductivity and stacked structure. Herein, we report the rational assembly of a series of transition metal hydroxides on graphene to act as a cocatalyst ensemble for efficient CO_2_ photoreduction. In particular, with the Ru-dye as visible light photosensitizer, hierarchical Ni(OH)_2_ nanosheet arrays-graphene (Ni(OH)_2_-GR) composites exhibit superior photoactivity and selectivity, which remarkably surpass other counterparts and most of analogous hybrid photocatalyst system. The origin of such superior performance of Ni(OH)_2_-GR is attributed to its appropriate synergy on the enhanced adsorption of CO_2_, increased active sites for CO_2_ reduction and improved charge carriers separation/transfer. This work is anticipated to spur rationally designing efficient earth-abundant transition metal hydroxides-based cocatalysts on graphene and other two-dimension platforms for artificial reduction of CO_2_ to solar chemicals and fuels.

## Abstract (web summary)

The development of effective, earth-abundant cocatalysts is critical for photocatalytic CO_2_ reduction. Here, authors report the assembly of transition metal hydroxides on graphene to act as cocatalyst ensembles for efficient CO_2_ photoreduction.

## Introduction

Artificial photoreduction of carbon dioxide (CO_2_) into useful chemical feedstocks offers a promising and sustainable long term solution to the issues of increasing energy demands and climate change^1–6^. Nevertheless, there is still a long way to achieve efficient and selective photocatalytic CO_2_ reduction, especially in diluted CO_2_, which is primarily because of the low CO_2_ adsorption, high recombination rate of charge carriers in photocatalysts, concealed active sites, and the competing H_2_ evolution reaction^7–11^. To overcome these limitations, rational construction of hybrid photocatalytic systems, in which photosensitizers and cocatalysts operate in a harmonious manner, has arisen as a promising approach^12–16^.

Recently, the earth-abundant transition metal hydroxides, especially Ni(OH)_2_, as cocatalysts for photocatalytic CO_2_ reduction, have attracted extensive research interests due to their low cost, easy preparation, and effective adsorption for CO_2_^2,17^. However, the charge transfer efficiency of Ni(OH)_2_ is inferior because of its inherent weakness of poor conductivity, which results in the net efficiency of Ni(OH)_2_ in improving the photoactivity is often limited^18,19^. An effective way to overcome this disadvantage could be incorporating Ni(OH)_2_ with electrically conductive graphene (GR)^1,20^. In addition, the two-dimensional (2D) GR nanosheets with a large π-conjugated structure can further enhance the adsorption of CO_2_ via interacting with delocalized π-conjugation binding ${\uppi}^{4}_{3}$ of CO_2_ molecule^21,22^. Moreover, the large 2D platform and amenable wet-chemistry processability of GR also benefit the controllable composite structure construction^1,23–25^. In particular, rationally constructing nanoarray gradient structures based on vertical sheets-on-sheets is potentially able to harmoniously exert the synergistic ensemble effects of accelerating separation/transfer of charge carriers and exposing open active sites for the efficient adsorption, activation, and photoreduction of CO_2_^18,26,27^.

Herein, we report the facile synthesis of a series of different transition metal hydroxides, including Ni(OH)_2_, Fe(OH)_3_, Cu(OH)_2,_ and Co(OH)_2_, onto the 2D platform of GR to act as cocatalysts ensemble for CO_2_ photoreduction to solar fuels. With the Ru-dye as a visible-light photosensitizer, the optimal hierarchical Ni(OH)_2_-10%GR nanosheet arrays composite exhibits superior activity and selectivity, achieving a high CO formation rate of 10725 µmol h^−1^ g^−1^ and selectivity of 96% in pure CO_2_. More importantly, even in diluted CO_2_ (10% CO_2_, representative CO_2_ concentration of waste gas from coal-fired power stations)^7^, this Ni(OH)_2_-10%GR composite still demonstrates the excellent performance with CO production rate of 7432 µmol h^−1^ g^−1^ and high selectivity of 92%, which significantly surpasses other counterparts including bare Ni(OH)_2_, Ni(OH)_2_ nanoparticles-graphene (Ni(OH)_2_ NPs-GR), Fe(OH)_3_-GR, Cu(OH)_2_-GR and Co(OH)_2_-GR and outperforms most of analogous hybrid cocatalysts system in literatures. The underlying origin for such superior visible-light photoactivity and selectivity over Ni(OH)_2_-10%GR has been credited for its appropriate synergy on the effective adsorption of CO_2_, enriched active sites for CO_2_ reduction, and excellent charge carriers separation and transfer. It is hoped that this work would provide instructive guideline for rational design of earth-abundant transition metal hydroxides-based cocatalysts by harnessing the rich surface chemistry of GR and other 2D materials platforms toward efficient and selective solar light driven CO_2_ reduction to value-added fuels and chemicals in a sustainable way.

## Results

### Synthesis and morphology

**Fig. 1:**
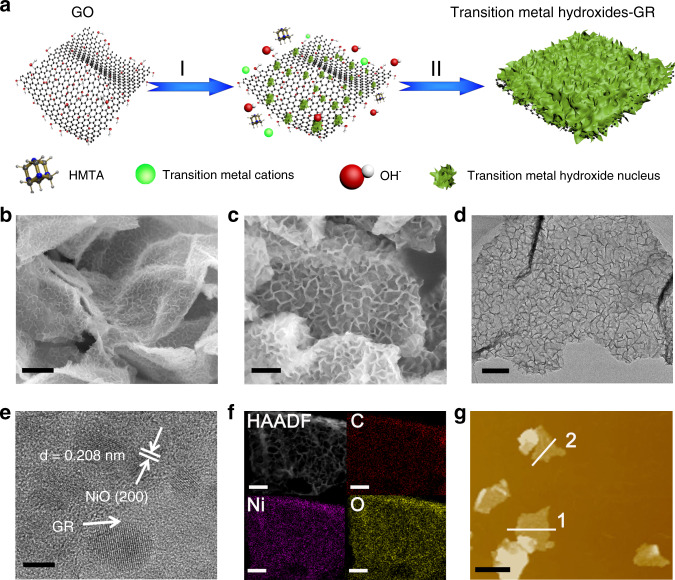
Synthesis and morphology characterization. **a** Schematic illustration of synthesis procedure for transition metal hydroxides onto the GR platform. I in situ heterogeneous nucleation, II oriented crystal growth. **b**, **c** FESEM images, **d** TEM image, **e** HRTEM image, **f** elemental mapping results, and **g** AFM image of the Ni(OH)_2_-10%GR. The scale bar are 500 nm in **b**, 200 nm in **c**, 100 nm in **d**, 2 nm in **e**, 1 µm in **f**, 3 µm in **g**.

As shown in Fig. 1a, the growth of transition metal hydroxides on the GR platform can be obtained via in situ heterogeneous nucleation and subsequent oriented crystal growth in solution phase. After transition metal precursor adding into the graphene oxide (GO) solution, the strong electrostatic interaction between negatively GO (Supplementary Fig. 1 and Supplementary Note 1) and transition metal cations (Ni^2+^, Fe^3+^, Cu^2+^, Co^2+^) leads to a firm adsorption of transition metal cations on the surface of GO^21^. At the beginning of the growth process, hexamethylenetetramine (HMTA) as a hydrolyzing agent can tardily decompose to liberate OH^–^ ions, and then transition metal cations react with OH^–^ ions to form dense transition metal hydroxides nuclei on the surface of GO^17,28^. The high density of transition metal hydroxide nuclei conduce to the vertical growth of nanosheets because the steric hindrance from adjacent seeds can hinder the in-plane direction growth^29^. Meanwhile, the GO precursor can also be reduced to GR during the thermal reflux process^1,20^, by which different transition metal hydroxide-GR composites are formed.

Since the Ni(OH)_2_-10%GR composite exhibits the optimal photocatalytic performance among the as-obtained transition metal hydroxide-GR composites as shown in the section of CO_2_ photoreduction, the following discussion will mainly focus on the characterizations of Ni(OH)_2_-10%GR composite. As shown in Supplementary Fig. 2 and Supplementary Note 2, the morphological evolution process of Ni(OH)_2_-10%GR has been investigated via time-dependent SEM images. It can be seen that GO surface is smooth before the refluxing reaction. As refluxing time increases, Ni(OH)_2_ gradually nucleates and grows on the GR surface, which makes GR surface become rough, and when the refluxing time is prolonged to 7 h, Ni(OH)_2_ nanosheet arrays structure begins to form on the GR surface. After refluxing for 10 h, the surface of GR is covered with homogeneous Ni(OH)_2_ nanosheet arrays as revealed by the SEM images in Fig. 1b, c. In addition, as shown in Supplementary Fig. 3, the thickness of the Ni(OH)_2_ layers in Ni(OH)_2_-10%GR composite can be found to be ca. 10 nm. Notably, it is pivotal to regulate the amount of GR to achieve such evenly dispersed hiearchical Ni(OH)_2_ nanosheet arrays on the GR platform because the amount of GR affects the morphology of Ni(OH)_2_-GR hybrids significantly. As shown in Supplementary Fig. 4, when the amount of GR is 1 wt% or 5 wt%, stacked Ni(OH)_2_ nanosheet arrays are obtained whereas nanosheet array structures cannot be formed when the amount of GR is 30 wt% or 50 wt%. In addition, in the absence of GR, the as-obtained blank Ni(OH)_2_ displays a sphere-like aggregated structure (Supplementary Fig. 5), which is because the nucleus formation and growth of blank Ni(OH)_2_ lack steric hindrance, thereby leading to an omnidirectional and superimposed assembly^23^.

As shown in Fig. 1d, the hierarchical nanosheet array structure of Ni(OH)_2_-10%GR is further confirmed by transmission electron microscopy (TEM) characterization. In addition, as shown in Fig. 1e and Supplementary Fig. 6, because the α-Ni(OH)_2_ is sensitive to electron-beam irradiation, only high-resolution TEM image of NiO evolved from the α-Ni(OH)_2_ has been caught (Supplementary Note 3)^30–33^. Furthermore, elemental mapping images of Ni(OH)_2_-10%GR hybrid in Fig. 1f show the spatial distributions of C, O, and Ni, indicating the uniform growth of Ni(OH)_2_ nanosheet arrays on the matrix of GR. Furthermore, to determine the length of Ni(OH)_2_ nanosheet arrays, atomic force microscopy (AFM) of the Ni(OH)_2_-10%GR hybrid has been conducted. As displayed in Fig. 1g and Supplementary Fig. 7, the topography of Ni(OH)_2_-10%GR composite shows a 2D lamellar structure with a thickness of *ca*. 60 nm. Considering the thickness of GR sheet (0.9 nm)^34^ and the growth of Ni(OH)_2_ on both sides of GR, the length of Ni(OH)_2_ nanosheet arrays is deduced to be about 30 nm.

### Structure characterization

**Fig. 2:**
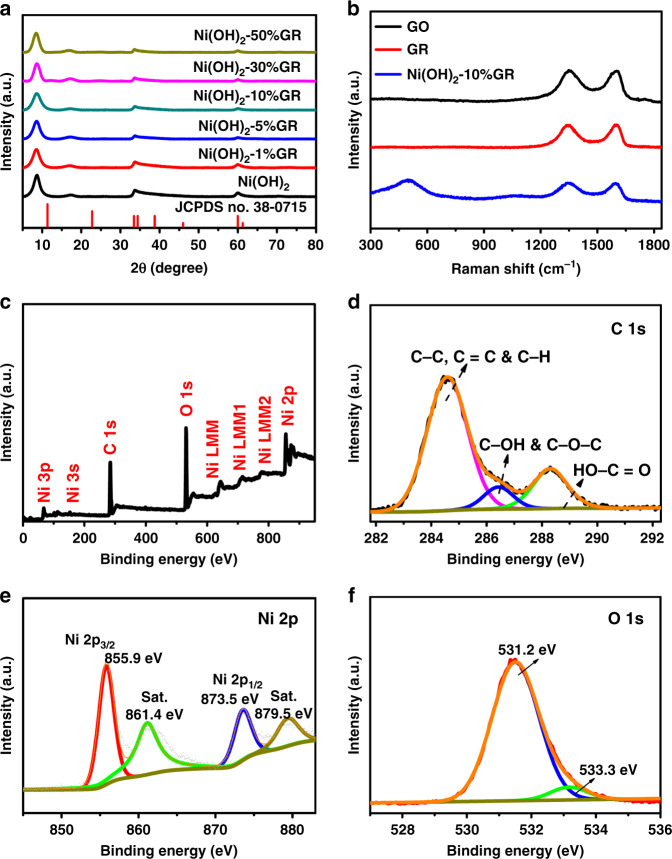
Structure characterization of Ni(OH)_2_-10%GR. **a** XRD patterns of Ni(OH)_2_-GR composite with different GR contents and bare Ni(OH)_2_. **b** Raman spectra of GO, GR and Ni(OH)_2_-10%GR. **c** XPS survey spectrum, high-resolution XPS spectra of **d** C 1 s, **e** Ni 2p, and **f** O 1 s of Ni(OH)_2_-10%GR.

X-ray diffraction (XRD) was used to characterize the crystal structures of these as-synthesized samples. As shown in Fig. 2a, the diffraction peaks of Ni(OH)_2_ at 8.7°, 17.3°, 33.4°, and 59.9° can be indexed to (003), (006), (101), and (110) planes of α-Ni(OH)_2_ (JCPDS no. 38-0715)^28,35^. Two peaks at low angles (<20°) clearly indicate the layered structure of Ni(OH)_2_^32^. Notably, the positions of (003) and (006) diffraction peaks shift towards lower angle as compared to that of standard card, indicating enlarged interlayer spacing of as-obtained Ni(OH)_2_, which has been widely reported in the previous literatures^32,36,37^. In addition, Ni(OH)_2_-GR hybrids exhibit analogous XRD patterns to that of the blank Ni(OH)_2_. The diffraction peaks of GR (Supplementary Fig. 8) have not been observed in these composites, which could be because GR layers are densely wrapped by Ni(OH)_2_ nanosheet arrays^18,23^. The analysis of Raman spectroscopy in Fig. 2b shows that peaks at 1353 and 1590 cm^−1^ are assigned to the D-band and G-band of GR, and the peak at 468 cm^−1^ is ascribed to Ni(OH)_2_^28,38^. The well-identified Ni(OH)_2_ and GR peaks in Ni(OH)_2_-10%GR composite indicate that Ni(OH)_2_ nanosheet arrays have successfully grown on the GR platform. Notably, the *I*_D_/*I*_G_ ratio is 0.97 for Ni(OH)_2_-10%GR, which is lower than 1.01 for GO, indicating that the thermal reduction process enhances the graphitization of GR^34^. Chemical composition and elemental states of Ni(OH)_2_-10%GR composite have been further monitored by X-ray photoelectron spectroscopy (XPS). The survey XPS spectrum in Fig. 2c confirms the existing elements of C, Ni and O in Ni(OH)_2_-10%GR composite. In addition, compared with C 1s spectrum of original GO in Supplementary Fig. 9, the C 1s spectrum of Ni(OH)_2_-10%GR hybrid shows an obvious loss of oxygenated functional groups, confirming the effective reduction of GO to GR during the thermal reflux process (Fig. 2d)^39,40^. Furthermore, in the Ni 2p region of Ni(OH)_2_-10%GR composite (Fig. 2e), peaks of Ni 2p_3/2_ and Ni 2p_1/2_ are at 855.9 and 873.5 eV, which indicates that the Ni species are in +2 valence state^17,35,41^. In addition, two shoulder peaks at 861.4 and 879.5 eV are related to the satellite peaks of Ni 2p_3/2_ and Ni 2p_1/2_^7^. The O 1 s spectrum in Fig. 2f displays a typical peak at 531.2 eV, which can be ascribed to the Ni-OH bond in Ni(OH)_2_-10%GR composite^18^. The peak situated at 533.3 eV is assigned to the adsorbed H_2_O molecules^30,42^. Moreover, as shown in Supplementary Fig. 10 and Supplementary Note 4, the thermogravimetric (TG) analysis demonstrates that the actual content of GR in the Ni(OH)_2_-10%GR hybrid is ca. 10.3 wt%, which almost equals to the feedstock proportion.

### Photoreduction CO_2_ performance

**Fig. 3:**
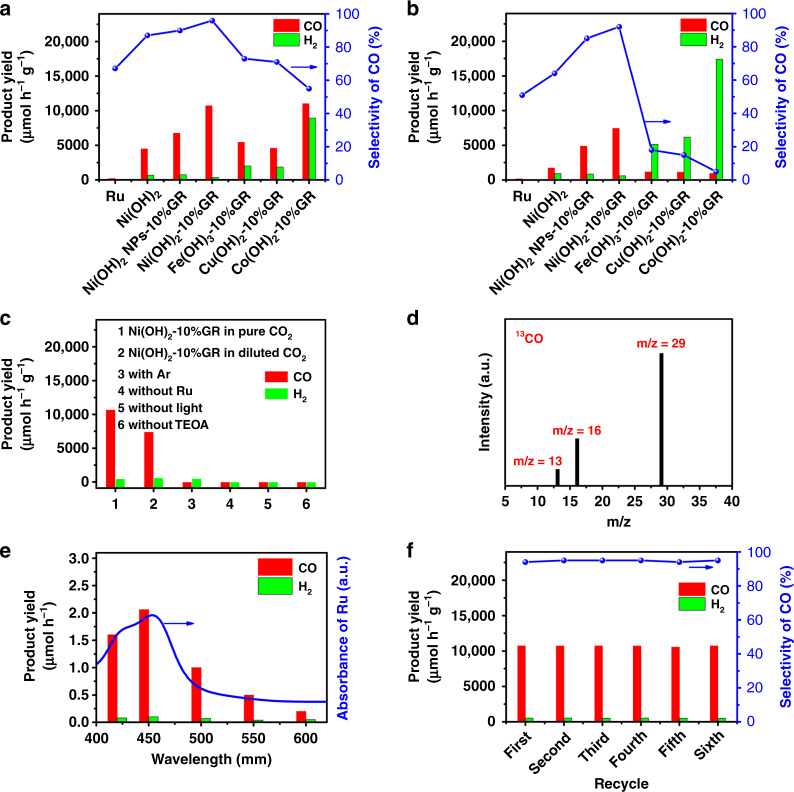
The photocatalytic performance of CO_2_ reduction. CO_2_ photoreduction performance over Ru, Ni(OH)_2_, Ni(OH)_2_ NPs-10%GR, Ni(OH)_2_-10%GR, Fe(OH)_3_-10%GR, Cu(OH)_2_-10%GR, and Co(OH)_2_-10%GR **a** in pure CO_2_ and **b** in diluted CO_2_. **c** CO_2_ photoreduction performance under various reaction conditions. **d** Mass spectrum of ^13^CO (m/z = 29) produced over Ni(OH)_2_-10%GR in the photocatalytic reduction of ^13^CO_2_. **e** Wavelength dependence of the yields of CO and H_2_ over Ni(OH)_2_-10%GR, and the light absorption spectrum of the Ru photosensitizer. **f** Recycling test of Ni(OH)_2_-10%GR.

The visible-light-driven photocatalytic CO_2_ reduction reactions were performed in a catalytic system with [Ru(bpy)_3_]Cl_2_·6H_2_O (abbreviated as Ru) as photosensitizer and triethanolamine (TEOA) as electron donor. Acetonitrile (MeCN) is selected as a reaction solvent because of its high solubility for CO_2_. CO and H_2_ are detected as the main gas phase products and no liquid phase products (*e.g*., HCHO, CH_3_OH, and HCOOH) are detected (Supplementary Fig. 11 and Supplementary Note 5). As displayed in Fig. 3a, in the presence of Ru alone, low CO evolution rate (187 µmol h^−1^ g^−1^) and selectivity (67%) are achieved in pure CO_2_, which is due to the poor charge transfer efficiency and lack of surface active sites^8,43,44^. The addition of Ni(OH)_2_ cocatalyst into the Ru solution leads to a significant improvement of photocatalytic performance. The rate of evolved CO reaches a value of 4492 µmol h^−1^ g^−1^ with CO selectivity of 87%, which indicates that Ni(OH)_2_ is an effective cocatalyst for photocatalytic CO_2_ reduction. Further improvements of photocatalytic activity and selectivity have been achieved after compositing Ni(OH)_2_ nanosheet arrays with electrically conductive GR. As shown in Supplementary Fig. 12 and Supplementary Note 6, when the content of GR is 10%, the Ni(OH)_2_-10%GR composite shows the optimal photocatalytic performance, achieving the CO formation rate of 10725 µmol h^−1^ g^−1^, which is ~2.3 and 57.2 times as high as that of blank Ni(OH)_2_ and bare Ru, respectively. The apparent quantum efficiency (AQE) for CO formation at 450 nm is calculated to be 1.03%. In addition, the Ni(OH)_2_-10%GR hybrid exhibits a superior selectivity of 96% for photocatalytic CO_2_ to CO reduction. Moreover, as shown in Supplementary Table 1, it can be found that the photocatalytic activity and selectivity of Ni(OH)_2_-10%GR composite in pure CO_2_ has exceeded that of most recently reported cocatalysts. Furthermore, Ni(OH)_2_ nanoparticles-graphene (Ni(OH)_2_ NPs-10%GR) hybrid has also been synthesized for a comparative study (Supplementary Figs. 13–15 and Supplementary Note 7–9). As demonstrated in Fig. 3a, the Ni(OH)_2_ NPs-10%GR composite exhibits lower photocatalytic activity (CO formation rate of 6742 µmol h^−1^ g^−1^) and CO selectivity (90%) than that of the Ni(OH)_2_-10%GR hybrid, which indicates that constructing hierarchical nanosheet array structure is an effective way to improve photocatalytic performance of the transition metal hydroxide-GR composite.

To assess the practical application of the obtained catalysts, we further perform the CO_2_ reduction reaction in diluted CO_2_ (10% CO_2_, representative CO_2_ concentration of waste gas from coal-fired power stations)^45^. As demonstrated in Fig. 3b, Ni(OH)_2_-10%GR composite displays a superior photocatalytic activity with the CO formation rate of 7432 µmol h^−1^ g^−1^, corresponding to AQE of 0.95% at 450 nm. Significantly, Ni(OH)_2_-10%GR still exhibits a high CO selectivity of 92% in diluted CO_2_. As shown in Supplementary Table 2, the photocatalytic performance of Ni(OH)_2_-10%GR hybrid in diluted CO_2_ is also superior than that of most previously reported results regarding analogous hybrid photocatalyst system in literatures. In addition, the photocatalytic activity and selectivity of Ni(OH)_2_-10%GR hybrid are much higher than that of blank Ni(OH)_2_ (CO generation rate of 1717 µmol h^−1^ g^−1^ and selectivity of 64%) and Ni(OH)_2_ NPs-10%GR counterpart (CO formation rate of 4890 µmol h^−1^ g^−1^ and selectivity of 85%) in diluted CO_2_.

The photocatalytic performance of other transition metal hydroxide-GR (Fe(OH)_3_-GR, Cu(OH)_2_-GR and Co(OH)_2_-GR) composites have also been tested for comparison under identical reaction conditions (Supplementary Figs. 16–18, Supplementary Notes 10 and 11). Similar to Ni(OH)_2_-GR composites, Fe(OH)_3_-GR, Cu(OH)_2_-GR, and Co(OH)_2_-GR composites also exhibit the optimal photocatalytic performance when the content of GR is 10%. As displayed in Fig. 3a, b, Ni(OH)_2_-10%GR or Ni(OH)_2_ NPs-10%GR composite shows obviously higher photoactivity and selectivity than that of Fe(OH)_3_-10%GR and Cu(OH)_2_-10%GR in both pure CO_2_ and diluted CO_2_. Notably, Co(OH)_2_-10%GR shows a CO production rate of 11625 µmol h^−1^ g^−1^ in pure CO_2_, which is slightly higher than that of Ni(OH)_2_-10%GR. However, the CO selectivity over Co(OH)_2_-10%GR only reaches 56%. Moreover, the CO formation rate of Co(OH)_2_-10%GR dramatically declines to 945 µmol h^−1^ g^−1^ with a low CO selectivity of 4% in diluted CO_2_. These results clearly demonstrate that Ni(OH)_2_ is a more efficient and selective cocatalyst for photocatalytic reduction of CO_2_ to CO than other transition metal hydroxides.

To further study the factors that could influence the performance of photocatalysts, a series of control experiments are conducted. As displayed in column 3 of Fig. 3c, the comparative reaction in pure argon (Ar) indicates that only slight H_2_ is produced and no CO is formed. In addition, isotopic experiment with ^13^CO_2_ as substrate has also been carried out to confirm the origin of CO. As displayed in Fig. 3d, the mass spectrum signal of ^13^CO (m/z = 29) is clearly detected. These results definitely confirm that CO indeed derives from the photocatalytic reduction of CO_2_^46^. In addition, as shown in column 4–6 of Fig. 3c, control experiments in the absence of Ru or visible-light irradiation or TEOA result in the formation of a trace amount of CO and H_2_, which confirms that the CO_2_ reduction is driven by visible-light irradiation and the sacrificial agent is of great significance. Moreover, as shown in Fig. 3e, the wavelength dependency of CO production shows that the tendency of CO generation matches well with the absorption spectrum of Ru photosensitizer, further confirming that the photocatalytic CO_2_ reduction is indeed driven by the absorbed photon of Ru^47^. Furthermore, the stability of Ni(OH)_2_-10%GR cocatalyst has been evaluated by cycle experiments. The result in Fig. 3f shows that the photoactivity loss over Ni(OH)_2_-10%GR composite is negligible after six consecutive cycles. In addition, as shown in Supplementary Figs. 19 and 20, the results of XRD and XPS over fresh and used Ni(OH)_2_-10%GR show the same crystalline phase structure and element composition. SEM and TEM images of the used Ni(OH)_2_-10%GR sample in Supplementary Fig. 21 also indicate no obvious structural change after visible light illumination for 12 h. These results clearly demonstrate that Ni(OH)_2_-10%GR is a stable cocatalyst for photocatalytic CO_2_ reduction under present conditions. In addition, the turnover number (TON) for CO formation respect to Ru atoms is 4.8 over 5 h of stable operation, reflecting the catalytic nature of the reaction. Notably, this TON value is higher than that of previously reported ruthenium-nickel molecular photocatalysts^48,49^.

### Mechanism of enhanced photocatalytic performance

**Fig. 4:**
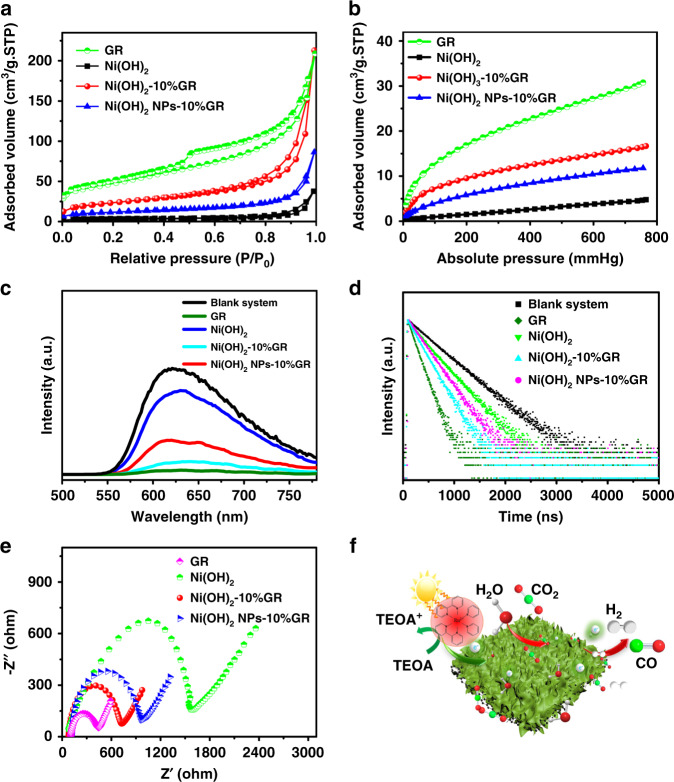
CO_2_ adsorption and photoelectrical properties of various photocatalysts. **a** N_2_ adsorption-desorption isotherms and **b** CO_2_ adsorption isotherms of GR, Ni(OH)_2_, Ni(OH)_2_ NPs-10%GR and Ni(OH)_2_-10%GR. **c** Steady-state PL spectra and **d** time-resolved PL spectra decay of the photocatalytic CO_2_ reduction systems with cocatalysts of GR, Ni(OH)_2_, Ni(OH)_2_ NPs-10%GR, Ni(OH)_2_-10%GR and without cocatalysts. **e** EIS Nyquist plots of GR, Ni(OH)_2_, Ni(OH)_2_ NPs-10%GR, and Ni(OH)_2_-10%GR. **f** Proposed photocatalytic mechanism of Ni(OH)_2_-10%GR with Ru photosensitizer for visible-light-driven photocatalytic CO_2_ reduction reaction.

To reveal the origin of the enhanced photocatalytic performance of Ni(OH)_2_-10%GR composite compared to that of other counterparts, various complementary characterizations have been conducted. Firstly, the surface areas and CO_2_ uptake ability of these samples have been studied. As shown in Fig. 4a and Supplementary Table 3, the BET surface area of Ni(OH)_2_-10%GR hybrid (83 m^2^ g^−1^) is obviously higher than that of blank Ni(OH)_2_ (11 m^2^ g^−1^) and Ni(OH)_2_ NPs-10%GR composite (40 m^2^ g^−1^), which clearly indicates that introducing GR into the composite to constructe hierarchical nanosheet array structure is beneficial for increasing the specific surface area. The augmented surface area of Ni(OH)_2_-10%GR composite indicates an increased exposed surface active sites and effective mass transportation of reactants and products, which are conducive to enhance the photocatalytic activity of the composite^17,23^. In addition, the CO_2_ adsorption isotherms of GR, Ni(OH)_2_, Ni(OH)_2_-10%GR, and Ni(OH)_2_ NPs-10%GR composite have also been collected. As shown in Fig. 4b, it can be seen that GR has a strong adsorption capacity for CO_2_. After compositing Ni(OH)_2_ with GR, Ni(OH)_2_-10%GR and Ni(OH)_2_ NPs-10%GR composites exhibit significantly improved CO_2_ adsorption capacity compared to that of blank Ni(OH)_2_. Notably, the Ni(OH)_2_-10%GR hybrid shows higher CO_2_ adsorption capacity than that of Ni(OH)_2_ NPs-10%GR hybrid. Similar trends are also observed in CO_2_ temperature-programmed desorption (TPD) tests, as shown in Supplementary Fig. 22. These results suggest that the introduction of GR and construction of hierarchical nanosheet array structures can effectively enhance CO_2_ adsorption capacity of the composite, which is desirable to facilitate the enrichment and activation of CO_2_ molecule, thus contributing to the boosted photocatalytic performance of Ni(OH)_2_-10%GR composite for CO_2_ reduction^12,44^.

On the other hand, photoluminescence (PL) and electrochemical impedance spectroscopy (EIS) measurements have been conducted to explore the separation and transfer process of charge carriers, which are another crucial factor affecting the photoactivity of CO_2_ reduction^7,44^. As shown in Fig. 4c, d and Supplementary Table 4, the cocatalysts contained systems show much lower PL intensities and shorter PL lifetimes than that of blank system. Generally, the lower PL emission intensity and shorter PL lifetime indicate the more efficient suppression of photoexcited charge recombination^8,50^. In addition, as shown in Supplementary Fig. 23, there is almost no influence on the light absorption of Ru by the addition of Ni(OH)_2_-10%GR cocatalyst, which further confirms that the quenching of PL intensity should be directly caused by the promoted transfer of photoexcited electrons^51^. Therefore, the introduction of cocatalysts can effectively promote the separation of charge carriers in the CO_2_ photoreduction system. Notably, compared with blank Ni(OH)_2_ and Ni(OH)_2_ NPs-10%GR contained systems, Ni(OH)_2_-10%GR contained system exhibits significantly lower PL intensity and shorter PL lifetime, which validates that introduction of conductive GR into the composite for constructing hierarchical nanosheet array structures is able to effectively facilitate the separation of photogenerated charge carriers. In addition, as revealed in Fig. 4e, the EIS spectrum of Ni(OH)_2_-10%GR displays a smaller semicircle in the Nyquist plot than that of blank Ni(OH)_2_ and Ni(OH)_2_ NPs-10%GR composite, which further confirms the elevated conductivity of the hybrid and the superiority of hierarchical nanosheet array structure in promoting the transfer of photogenerated charge carriers^52,53^.

Previously reported theoretical calculations indicate that Ni sites possess stronger CO_2_ adsorption affinity and weaker H^+^ affinity than that of other transition metal sites^7,8,52^, which could be the main reason for the enhanced photocatalytic performance of Ni(OH)_2_-10%GR composite compared to other transition metal hydroxide-GR composites (Fe(OH)_3_-10%GR, Cu(OH)_2_-10%GR, and Co(OH)_2_-10%GR). As shown in Supplementary Fig. 24a, b, the enhanced CO_2_ adsorption capacity of Ni(OH)_2_-10%GR composite is confirmed by CO_2_ adsorption isotherm and CO_2_ TPD test. Notably, the Co(OH)_2_-10%GR composite shows the most effective charge separation and transfer among these samples as reflected by EIS and PL spectra analysis (Supplementary Fig. 24c–e and Supplementary Table 5). However, because of the lowest CO_2_ adsorption capacity of Co(OH)_2_-10%GR composite (Supplementary Fig. 24a, b), its photocatalytic performance is inferior in diluted CO_2_, which further confirms that CO_2_ adsorption capacity is the crucial factor for affecting the photocatalytic performance of the catalysts.

The Mott-Schottky measurements in Supplementary Fig. 25a–c show that flat band potentials of Ni(OH)_2_, GR, Ni(OH)_2_-10%GR are ca. −0.7 V, − 0.82 V and −0.76 V vs. normal hydrogen electrode (NHE), which are lower than that of E(Ru(bpy)_3_^2+^*/Ru(bpy)_3_^+^) = −1.09 V (vs. NHE) and higher than the redox potential of E(CO_2_/CO) = −0.53 V (vs. NHE)^54,55^. Hence, Ni(OH)_2_, GR and Ni(OH)_2_-10%GR all have suitable redox potential and can receive electrons from the reduced Ru complex to drive the photocatalytic CO_2_-to-CO reduction reaction. In addition, the energy level diagram illustrated in Supplementary Fig. 25d indicates that the fermi level (E_F_) value of GR is lower than the lowest unoccupied molecular orbital (LUMO) value of [Ru(bpy)_3_]Cl_2_ and higher than the E_F_ value of Ni(OH)_2_ (Supplementary Note 12). Therefore, the high carrier mobility and well-aligned energy levels of GR enable it to efficiently facilitate electron transfer from photosensitizers to Ni(OH)_2_^51,56^. On the basis of the above analysis, a possible reaction mechanism for the CO_2_ photoreduction over Ni(OH)_2_-10%GR composite has been proposed. As shown in Fig. 4f, under visible-light irradiation, the [Ru(bpy)_3_]^2+^ photosensitizer is activated to the excited state [Ru(bpy)_3_]^2+^*, which is then reductively quenched by TEOA, forming the reduced photosensitizer [Ru(bpy)_3_]^+^^44^. Meanwhile, TEOA is oxidized to diethanolamine and glycolaldehyde^57,58^. Subsequently, the reduced photosensitizer [Ru(bpy)_3_]^+^ transfers electrons to GR, which further relays the electron to Ni(OH)_2_. Finally, the adsorbed CO_2_ on the Ni(OH)_2_ surface are activated and reduced to CO. At the same time, parts of excited electrons are accepted by protons to produce H_2_^7,55^. The strong CO_2_ affinity and weak H^+^ affinity of Ni sites endow Ni(OH)_2_ with great potential for CO_2_ uptake, thus promoting CO_2_-to-CO conversion and inhibiting the generation of H_2_ byproduct. In addition, compositing Ni(OH)_2_ with GR to construct hierarchical nanosheet array structures can synergistically expose abundant active sites for photocatalytic CO_2_ reduction, improve CO_2_ adsorption and promote separation and transfer of charge carriers. Therefore, Ni(OH)_2_-10%GR composite displays the optimal photocatalytic CO_2_ reduction performance as compared to that of other counterparts, including bare Ni(OH)_2_, Ni(OH)_2_ NPs-GR, Fe(OH)_3_-GR, Cu(OH)_2_-GR and Co(OH)_2_-GR.

## Discussion

In summary, we have rationally synthesized a series of transition metal hydroxides, including Ni(OH)_2_, Fe(OH)_3_, Cu(OH)_2,_ and Co(OH)_2_, on the GR platform to act as cocatalysts for artificial photoreduction of CO_2_. Specifically, hierarchical Ni(OH)_2_ nanosheet arrays-graphene (Ni(OH)_2_-GR) composites exhibit superior photocatalytic activity and selectivity. With the Ru-dye visible-light photosensitizer, the optimal Ni(OH)_2_-10%GR composite exhibits a CO formation rate of 10725 µmol h^−1^ g^−1^ with selectivity of 96%. Even in the diluted CO_2_, Ni(OH)_2_-10%GR composite still exhibits excellent photoactivity with the CO generation rate of 7432 µmol h^−1^ g^−1^ and high selectivity of 92%, which is markedly higher than that of other counterparts. A series of complementary characterizations suggest that the synergy of enhanced CO_2_ adsorption capacity, increased surface active sites, and improved charge separation/transfer of Ni(OH)_2_-10%GR composite leads to its boosted optimal performance for photocatalytic CO_2_ reduction as compared to that of other counterparts. It is expected that our current work could exploit new frontiers for the rational construction of earth-abundant transition metal hydroxides-based cocatalysts on graphene and other 2D material platforms for efficient and selective photoreduction of CO_2_ to value-added chemical feedstocks.

## Methods

### Materials

Hydrochloric acid (HCl), nickel nitrate hexahydrate (Ni(NO_3_)_2_·6H_2_O), triethanolamine (C_6_H_15_O_3_N, TEOA), sulfuric acid (H_2_SO_4_), cobalt nitrate hexahydrate (Co(NO_3_)_2_·6H_2_O), acetonitrile (CH_3_CN, MeCN), deuterium oxide (D_2_O), copper nitrate trihydrate (Cu(NO_3_)_2_·3H_2_O), hexamethylenetetramine (C_6_H_12_N_4_, HMTA), hydrogen peroxide (H_2_O_2_), iron nitrate nonahydrate (Fe(NO_3_)_3_·9H_2_O), potassium permanganate (KMnO_4_), trisodium citrate (C_6_H_5_O_7_Na_3_) and urea (CH_4_N_2_O) were supplied by Sinopharm Chemical Reagent Co., Ltd. (Shanghai, China). Cis-Dichlorobis(2,2-bipyridine)ruthenium(II) ([Ru(bpy)_2_]Cl_2_) were purchased form Sigma-Aldrich Co., Ltd (Shanghai, China). All chemicals were analytical grade and used as received. Deionized (DI) water was obtained from local sources.

### Catalyst preparation

*Synthesis of graphene oxide*: GO was synthesized by a modified Hummers’ method^34,59–62^. In detail, 10 g graphite powder (supplied from Qingdao Zhongtian Company, China) was put into 230 mL concentrated H_2_SO_4_ under moderate stirring. Then, 30 g KMnO_4_ was added gradually under stirring and the solution was cold below 5 °C in an ice bath. After that, the solution was heated to 35 °C in a water-bath and kept stirring for 2 h. Then, the mixture was diluted with 500 mL DI water in an ice bath to keep the temperature below 5 °C. Shortly after the further diluted with 1.5 L of DI water, 80 mL 30% H_2_O_2_ was then added into the mixture. The mixture was centrifuged and washed with 1:10 HCl aqueous solution to remove metal ions followed by DI water to remove the acid. After that, the mixture was dialyzed for one week and the final GO sample was obtained after full sonication.

*Synthesis of nickel hydroxide nanosheet arrays-graphene (Ni(OH)*_*2*_*-GR) composites*: firstly, a certain amount of GO was dispersed into 50 mL DI water with ultrasonication for 1 h. Then, 2.5 mmol Ni(NO_3_)_2_·6H_2_O was dissolved into the GO solution and stirred for 0.5 h. Afterward, a 50 mL solution containing 0.25 mmol of C_6_H_5_O_7_Na_3_ and 2.5 mmol of HMTA was incorporated to the above-mixed solution and stirred for 1 h. After that, the obtained solution was heated to 363 K and maintained for 10 h with vigorous agitation. Subsequently, when the solution temperature was cooled to 298 K, the sample was collected by centrifugation and washed thoroughly with DI water. After finally treated by freeze-drying, Ni(OH)_2_-GR composites with different contents of GR (1, 5, 10, 30, and 50 wt%) were obtained. Other transition metal hydroxide-graphene composites (Co(OH)_2_-GR, Fe(OH)_3_-GR and Cu(OH)_2_-GR) were prepared according to the same protocol as that for Ni(OH)_2_-GR composite except for using the same amount of the corresponding transition metal nitrate instead. In comparison, blank Ni(OH)_2_, Co(OH)_2_, Fe(OH)_3,_ and Cu(OH)_2_ were prepared using the identical process without GR platform. In addition, bare GR was prepared using the same method in the absence of transition metal nitrate.

*Preparation of nickel hydroxide nanoparticles-graphene (Ni(OH)*_*2*_ *NPs-GR) composite*: Ni(OH)_2_ NPs-10%GR composite was synthesized by a simple hydrothermal method^18,63^. Typically, 2 mmol Ni(NO_3_)_2_·6H_2_O, 6 mg GO and 250 mg of urea were dispersed into 30 mL DI water with ultrasonication for 1 h. After that, the mixed solution was added into a 50 mL Teflon-lined autoclave. The hydrothermal treatment was performed at 363 K for 10 h. When the temperature of solution was reduced to 298 K, the product was gathered by centrifugation, washed thoroughly with DI water, and treated by freeze-drying.

### Characterization

The structures of the samples were determined by dual beam SEM (Helios G4 CX) and TEM (FEI Tecnai G2 F20). AFM measurements were performed in Agilent 5500 AFM (Agilent Technologies, USA). XRD patterns were collected on a Rigaku Miniflex diffractometer with Cu Kα radiation. Thermogravimetric (TG) analysis was carried out on a PerkinElmer TGA7 analyzer under air atmosphere. BET surface areas and CO_2_ adsorption capacity of the as-prepared samples were evaluated by Micromeritics TriStar II PLUS 3020 equipment at 77 and 298 K, respectively. CO_2_ TPD measurements were performed on Micromeritics Auto Chem II 2920 instrument. XPS tests were executed on Thermo Scientific Escalab 250Xi spectrometer. Raman spectra were tested on a Renishaw inVia Raman System 1000 with a 532 nm Nd:YAG excitation source. Zeta-potentials (ζ) test was performed on Zetasizer 3000HSA. ^1^H nuclear magnetic resonance (^1^H NMR) measurement was conducted on Bruker DPX 400 spectrometer. Steady-state PL spectra and PL lifetime of these samples were tested on Edinburgh FLS-920 spectrofluorometer. The above PL measurements were performed in MeCN/H_2_O/TEOA (3:2:1, 6 mL) mixed solution containing 7.5 mg Ru-dye and 1 mg as-prepared cocatalysts. The excitation wavelength was 405 nm when steady-state PL was measured. For the test of PL lifetime, the light source was 405 nm laser and the emission wavelength was 630 nm. EIS and Mott-Schottky measurements were implemented on electrochemical workstation (Autolab PGSTAT204).

### Photocatalytic CO_2_ reduction testing

MeCN/H_2_O/TEOA (3:2:1, 6 mL) mixed solution containing 7.5 mg [Ru (bpy)_3_]Cl_2_·6H_2_O photosensitizer and 1 mg as-prepared cocatalysts was added into a gas-closed quartz reactor. Then, the quartz reactor was purged with CO_2_ (99.9999% or 10%) for 30 min. A 300 W Xe lamp (PLS-SXE300D, Perfectlight) with UV cutoff filter (*λ* ≥ 420 nm) was applied to as the light source and the light intensity was measured to be 405 mW cm^−2^. The reactor temperature was hold at room temperature by an electronic fan. For each 2 h, the gaseous products were analysed by gas chromatography (GC 2014C, Shimadzu). H_2_ was analyzed by a thermal conductivity detector (TCD). CO was converted to CH_4_ using a methanation reactor and then detected by a flame ionization detector (FID). The liquid products were analysed using ^1^H NMR. Isotope test was conducted by gas chromatography-mass spectrometry (GC-MS, 7890B and 5977 A, Agilent). The AQE was calculated according to the equation: AQE = [(2 × number of CO evolved molecules)/(number of incident photons)] × 100%. The turnover number (TON) for CO over Ru atoms was calculated using the following equation: TON = moles of CO evolved/moles of Ru atoms on photocatalyst. For recycling tests, the sample was collected and rinsed with DI water three times after 2 h of photocatalytic reaction. Then, the fresh reaction solution containing 7.5 mg Ru-dye was mixed with the used cocatalysts to conduct the second cycle experiment. By analogy, the subsequent four recycling tests were conducted.

## Supplementary information

supplementary information

## Data Availability

The data that support the findings of this study are available from the corresponding author on request. Source data are provided with this paper.

## Source data

Source Data
