# Supplementary material for: Rationally designed transition metal hydroxide nanosheet arrays on graphene for artificial CO2 reduction
Source: Nat Commun. 2020 Oct 14;11:5181. doi: 10.1038/s41467-020-18944-1 (PMC7560743; doi:10.1038/s41467-020-18944-1)
Supplement: Supplementary file 1 — supplementary information [file 41467_2020_18944_MOESM1_ESM.pdf]

Supporting Information

Rationally designed transition metal hydroxide nanosheet arrays on graphene for artificial CO_2 reduction

Authors: Lu *et al.*

Supplementary Figures

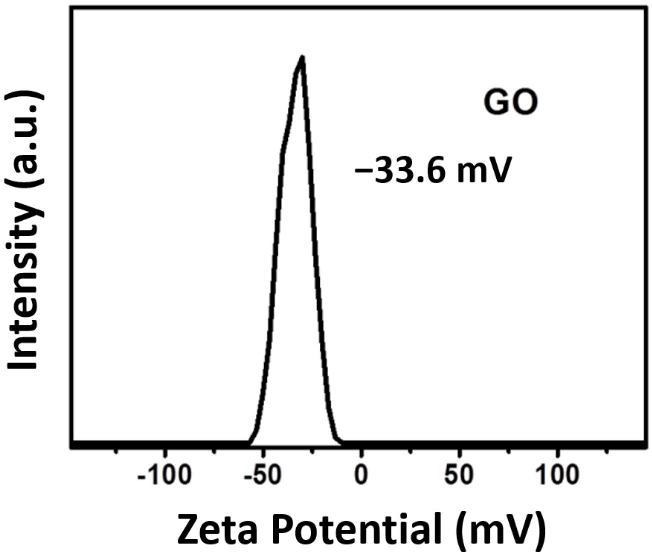

Supplementary Figure 1. Zeta potential of graphene oxide (GO) dispersed in deionized water.

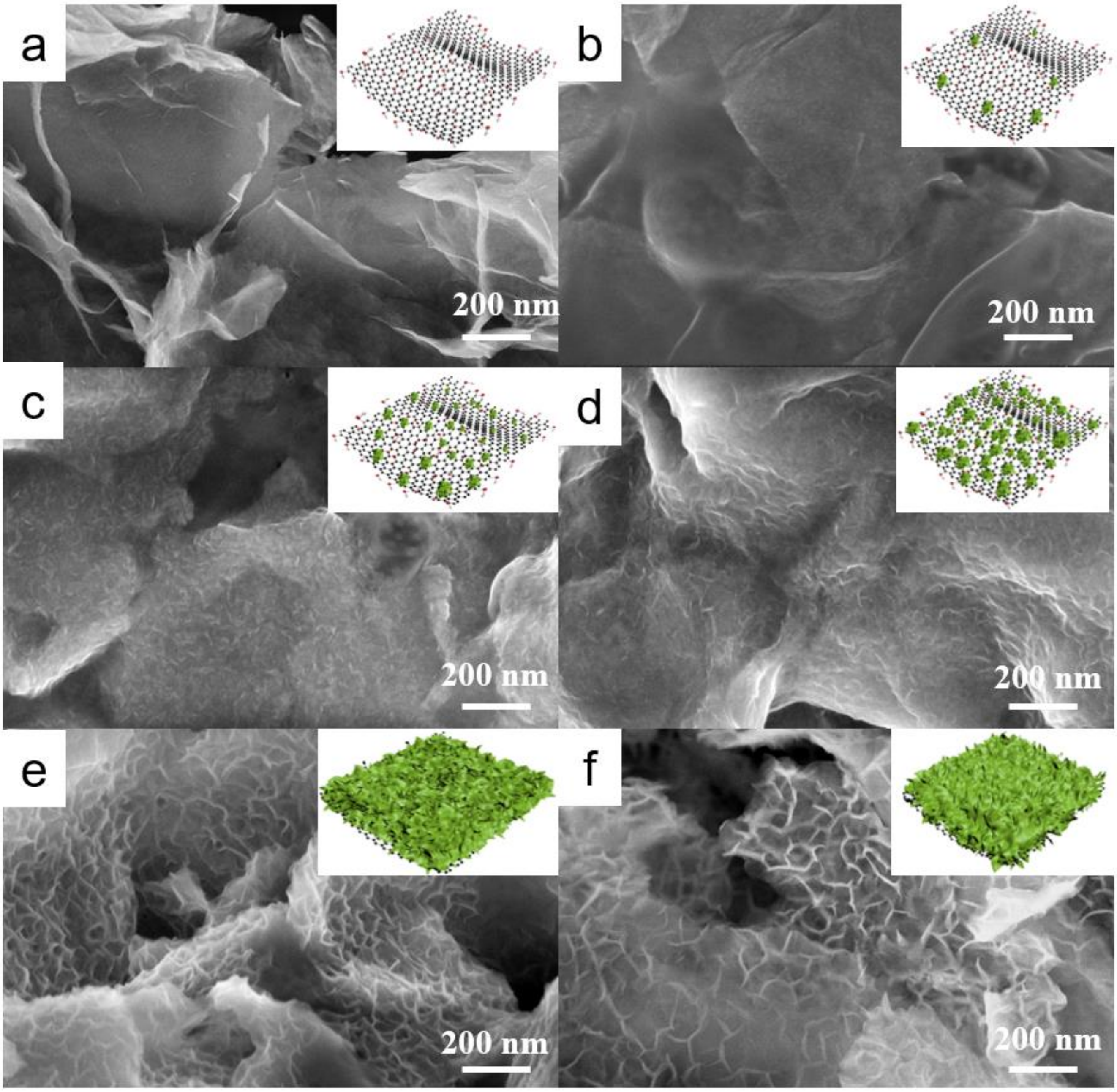

Supplementary Figure 2. Morphological evolution process of $Ni(OH)_2$-10%GR FESEM images of the $Ni(OH)_2$ nanosheet arrays grown on graphene platform prepared from $Ni(NO_3)_2 \cdot 6H_2O$, $C_6H_5O_7Na_3$ and HMTA at 90 ℃ for different refluxing time: (a) 0 h; (b) 1 h; (c) 3 h; (d) 5 h; (e) 7 h; (f) 9 h.

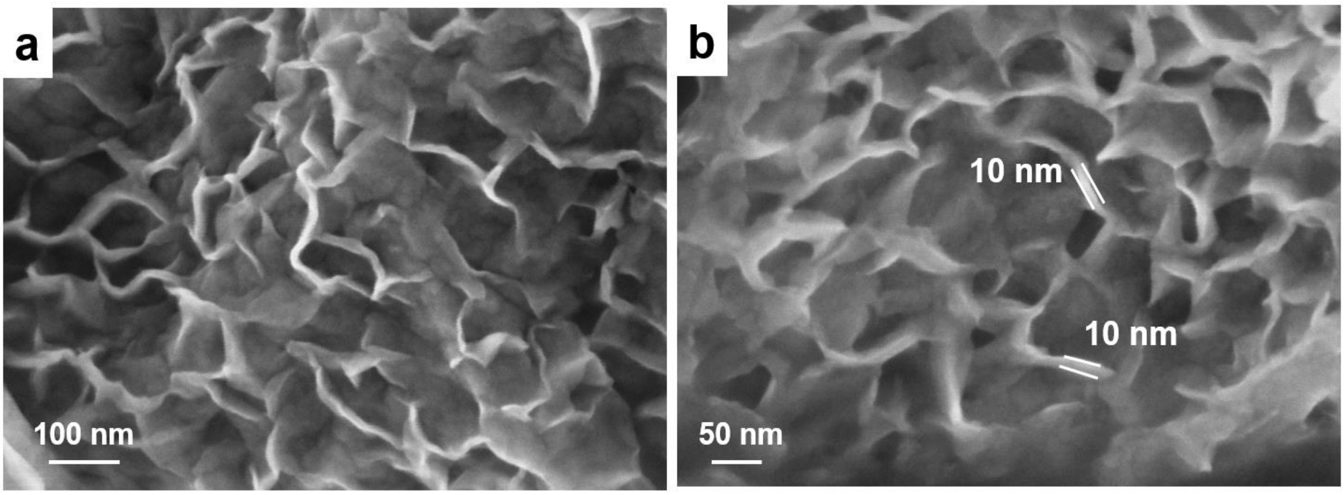

Supplementary Figure 3. Thickness information of $Ni(OH)_2$ FESEM images of $Ni(OH)_2$-10%GR with scale bar (a) 100 nm and (b) 50 nm.

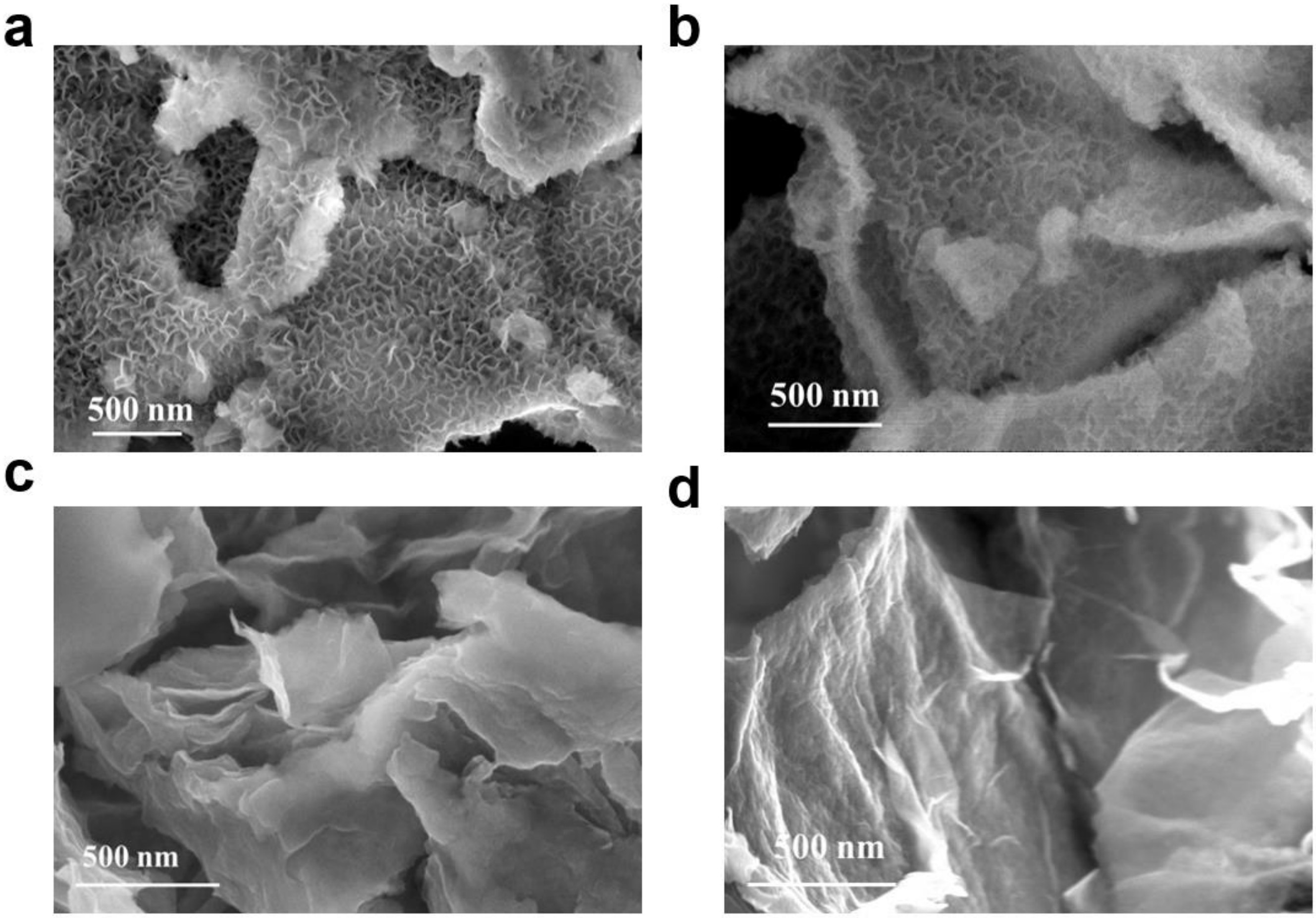

Supplementary Figure 4. Morphology information of $Ni(OH)_2$-GR FESEM images of (a) $Ni(OH)_2$-1%GR, (b) $Ni(OH)_2$-5%GR, (c) $Ni(OH)_2$-30%GR and (d) $Ni(OH)_2$-50%GR.

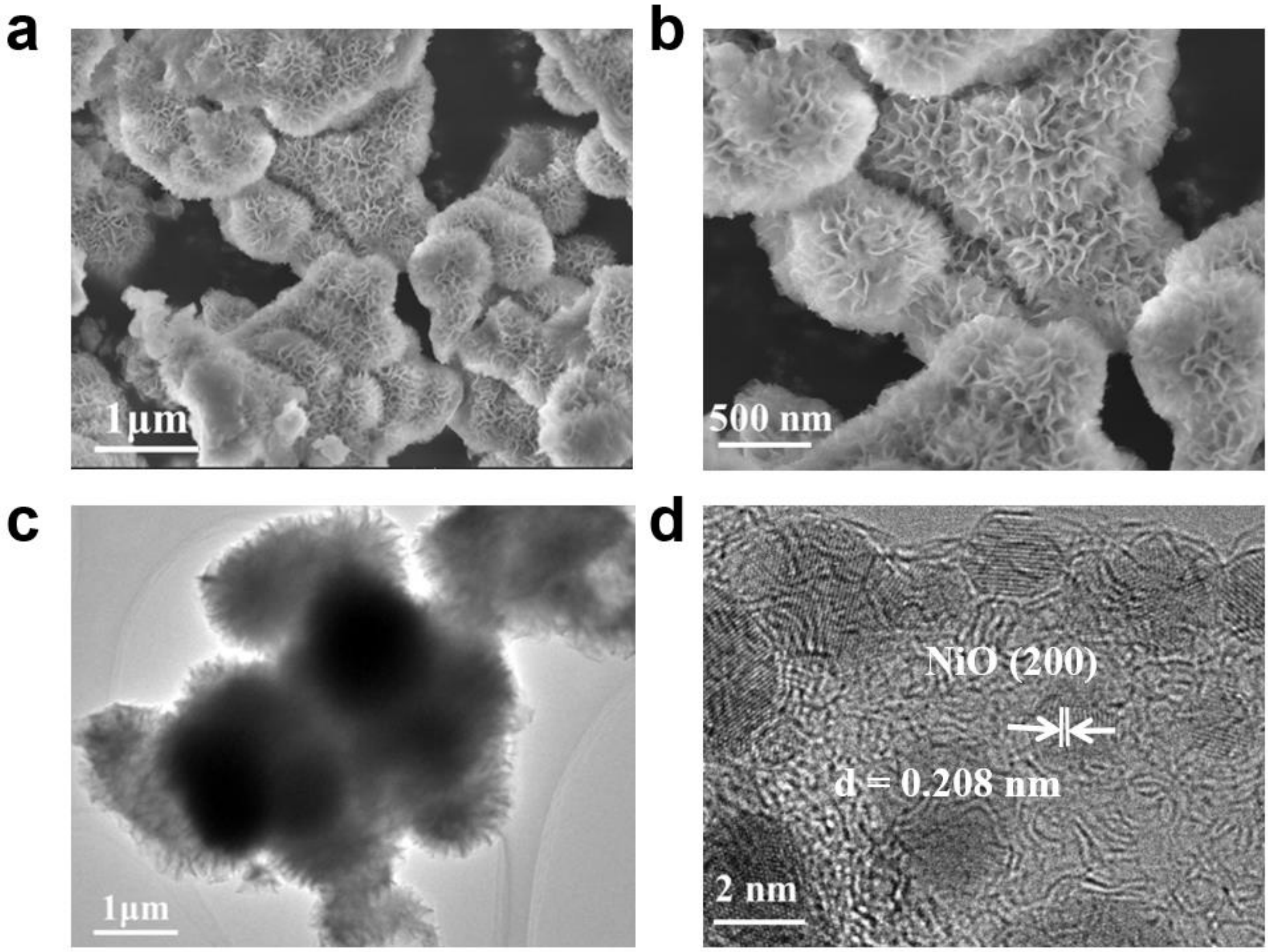

Supplementary Figure 5. Morphology information of $Ni(OH)_2$ (a, b) FESEM images, (c) TEM image and (d) HRTEM image of blank $Ni(OH)_2$.

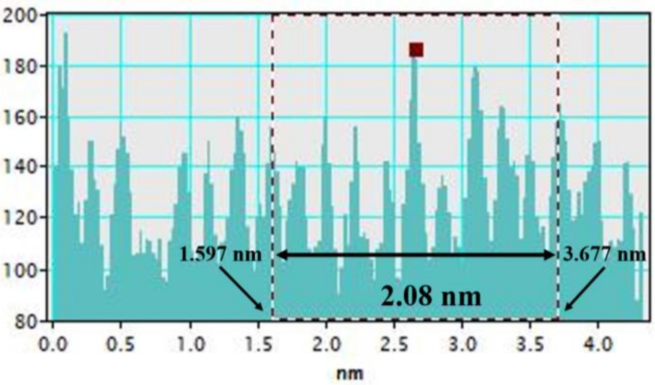

Supplementary Figure 6. Lattice distance measured by HRTEM of $Ni(OH)_2$-10%GR in Fig. 1e.

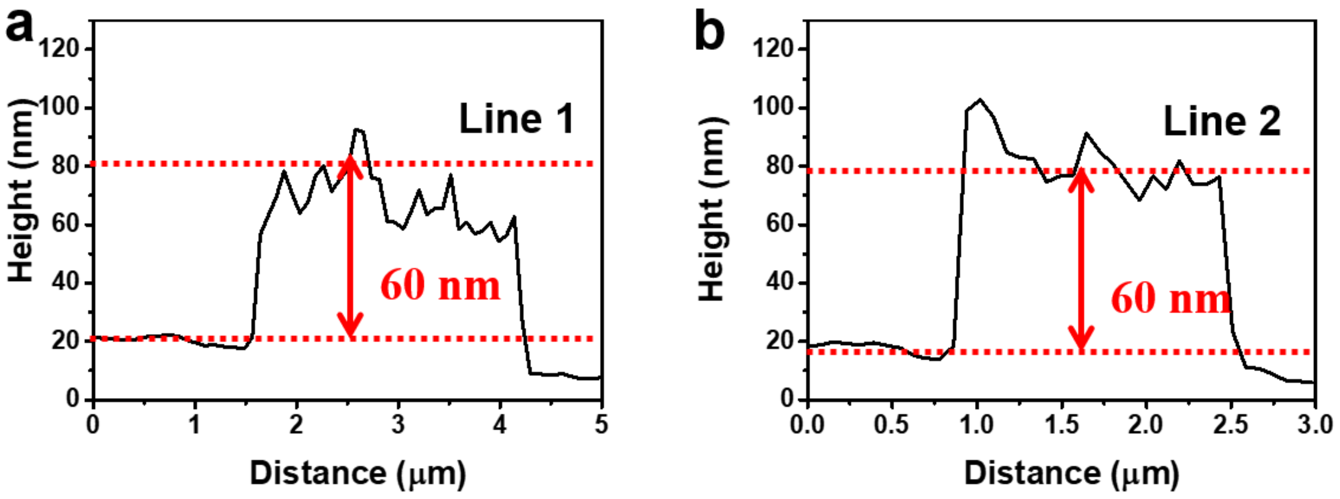

Supplementary Figure 7. Thickness information of $Ni(OH)_2$-10%GR The corresponding height profiles along the (a) white line 1 and (b) white line 2 drawn in Fig. 1g.

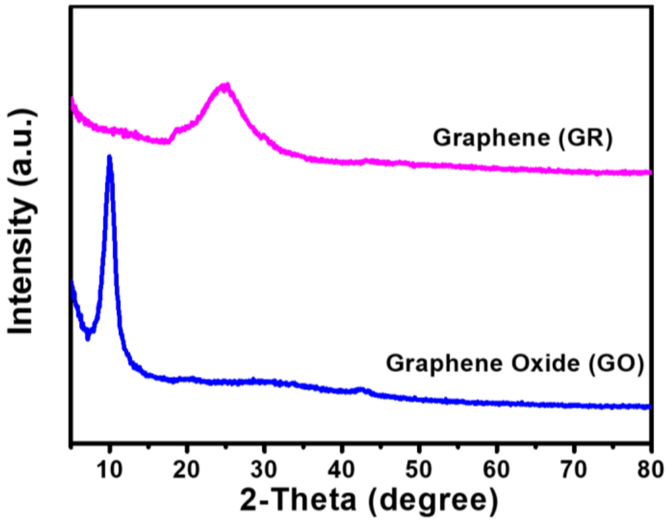

Supplementary Figure 8. XRD patterns of graphene oxide (GO) and graphene (GR).

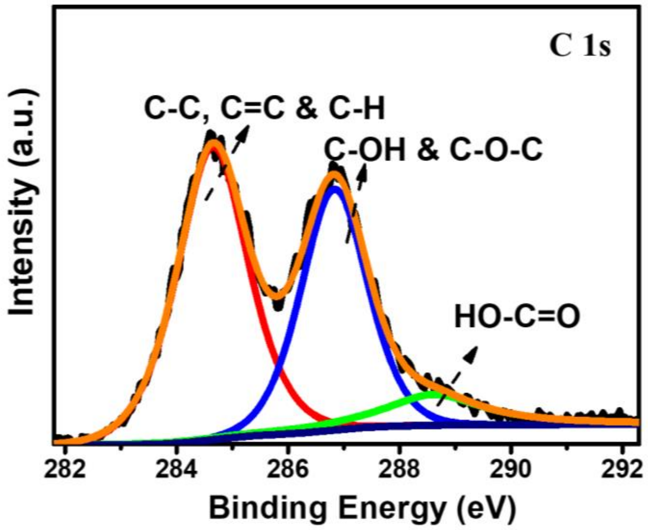

Supplementary Figure 9. High-resolution C 1s XPS spectrum of GO.

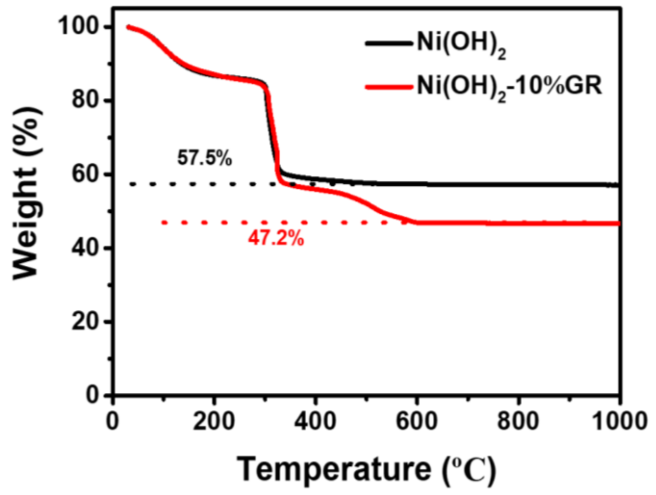

Supplementary Figure 10. **Thermogravimetric (TG) analysis** of blank $Ni(OH)_2$ and $Ni(OH)_2$-10%GR.

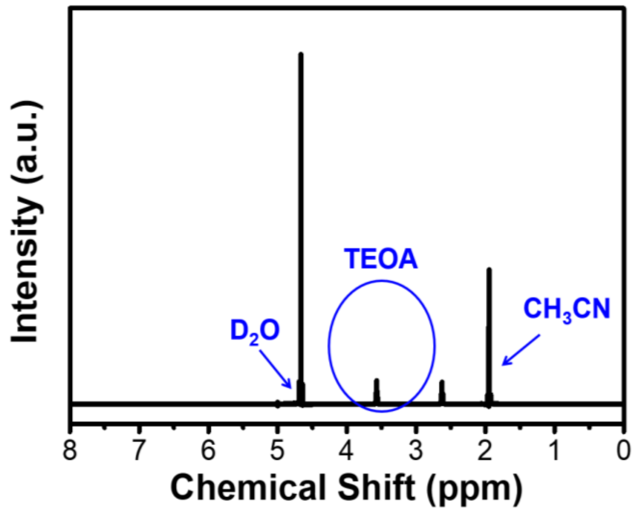

Supplementary Figure 11. ^{1}**H NMR spectrum** of the solution taken from the reaction system after visible-light irradiation for 2 h.

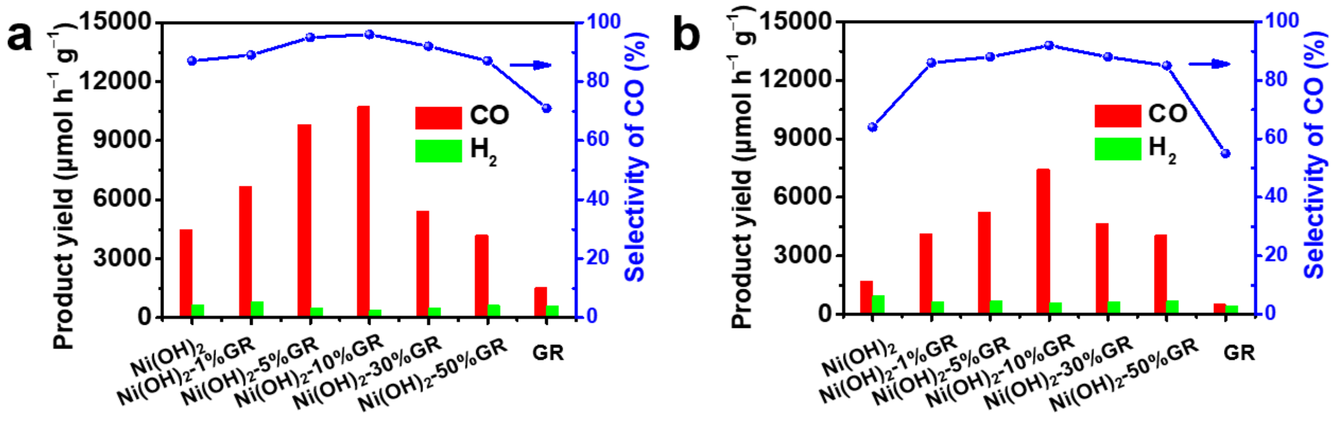

Supplementary Figure 12. CO_2 photoreduction performance over $Ni(OH)_2$-GR composites with different contents of GR in (a) pure CO_2 and (b) diluted CO_2.

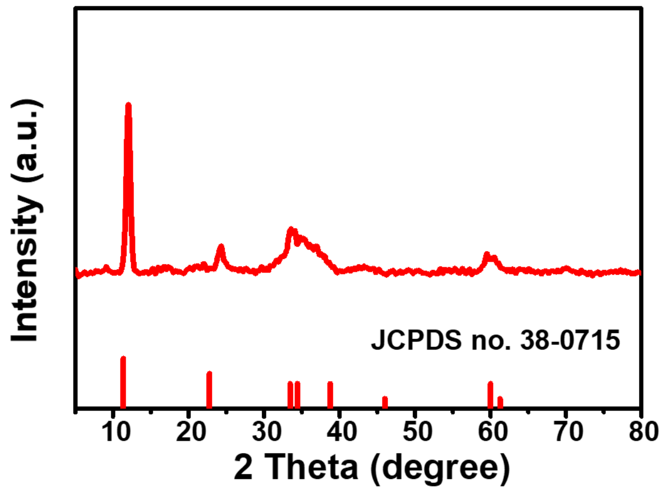

Supplementary Figure 13. XRD pattern of $Ni(OH)_2$ nanoparticles-graphene ($Ni(OH)_2$ NPs-10%GR).

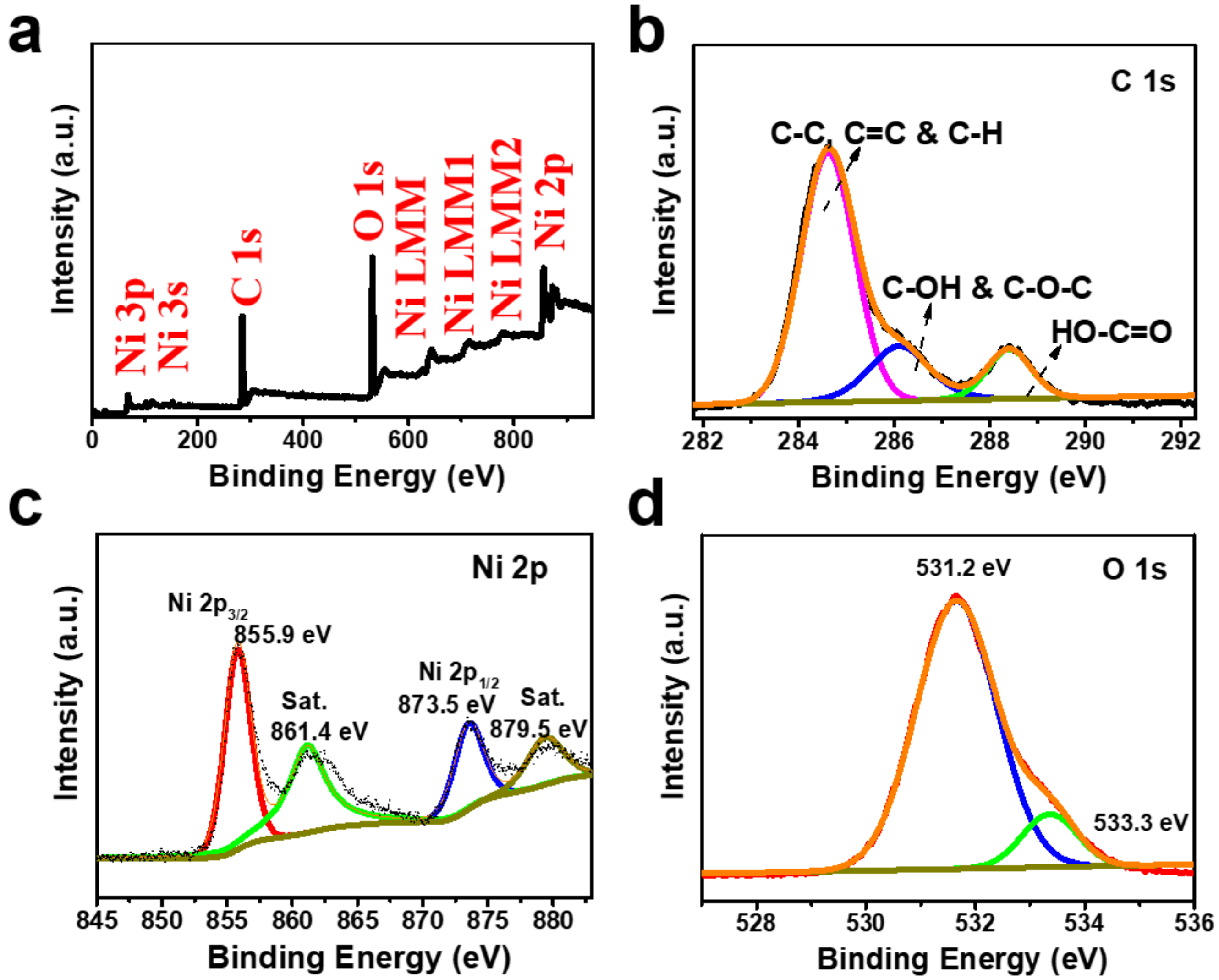

Supplementary Figure 14. XPS characterization of $Ni(OH)_2$ NPs-10%GR composite (a) XPS survey spectrum, (b) high-resolution XPS spectra of C 1s, (c) Ni 2p and (d) O 1s of $Ni(OH)_2$ NPs-10%GR composite.

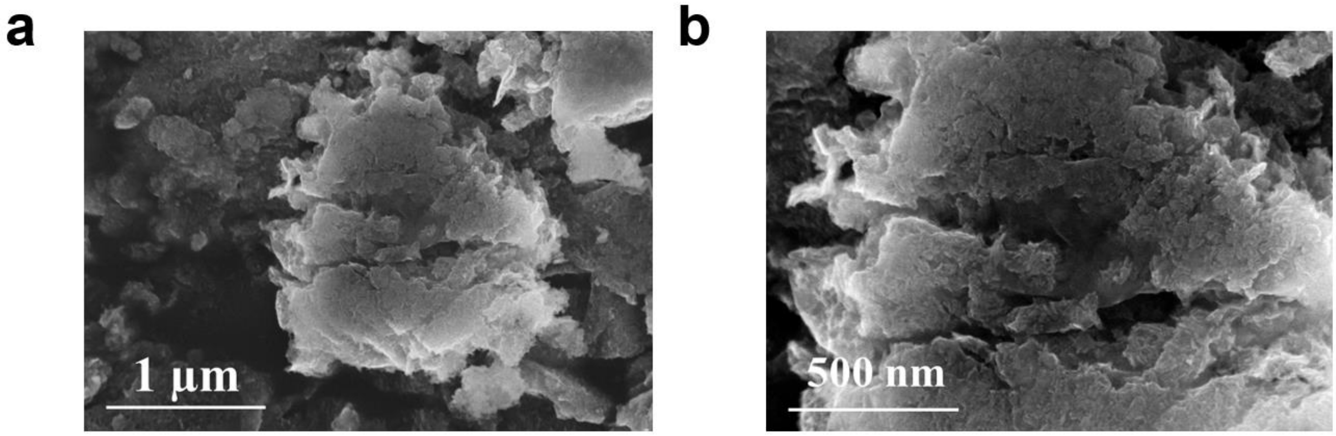

Supplementary Figure 15. Morphology information of $Ni(OH)_2$ NPs-10%GR composite FESEM images of $Ni(OH)_2$ NPs-10%GR composite with scale bar (a) 1 μm and (b) 500 nm.

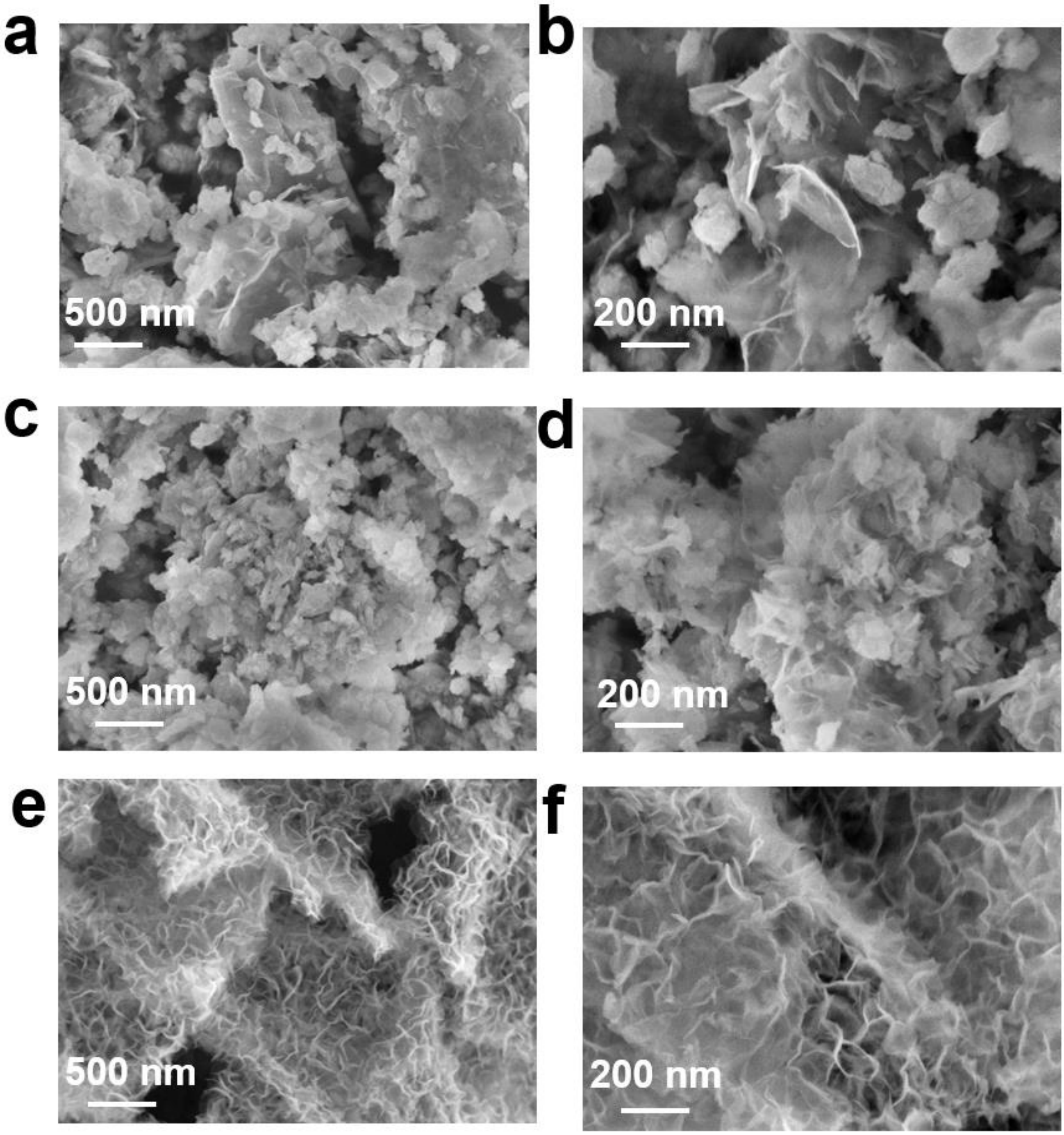

Supplementary Figure 16. Morphology information of as-obtained samples FESEM images of (a, b) $Fe(OH)_3$-10%GR, (c, d) $Cu(OH)_2$-10%GR and (e, f) $Co(OH)_2$-10%GR.

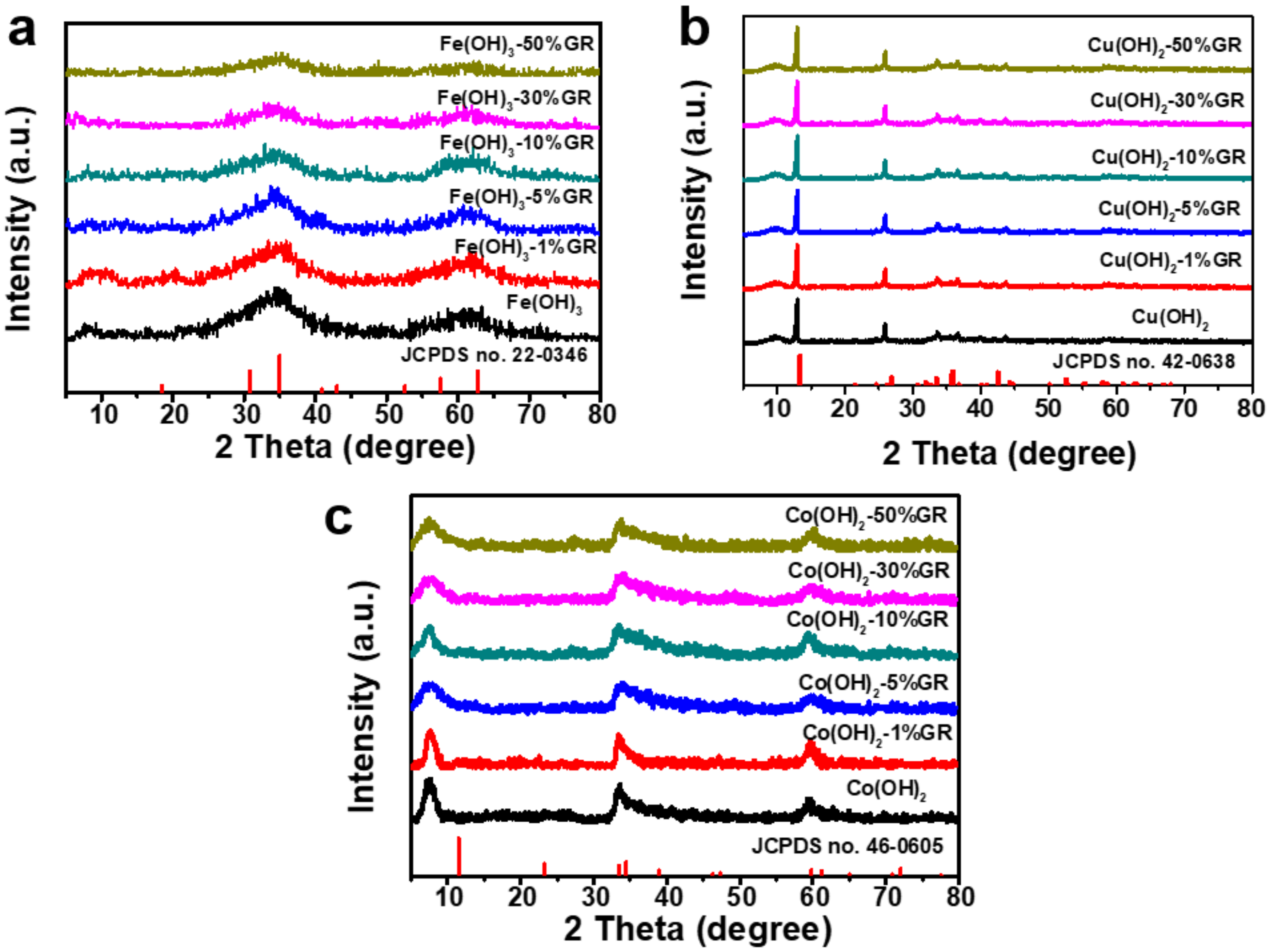

Supplementary Figure 17. XRD patterns of as-obtained samples (a) XRD patterns of $Fe(OH)_3$-GR with different GR contents and bare $Fe(OH)_3$. (b) XRD patterns of $Cu(OH)_2$-GR with different GR contents and bare $Cu(OH)_2$. (c) XRD patterns of $Co(OH)_2$-GR with different GR contents and bare $Co(OH)_2$.

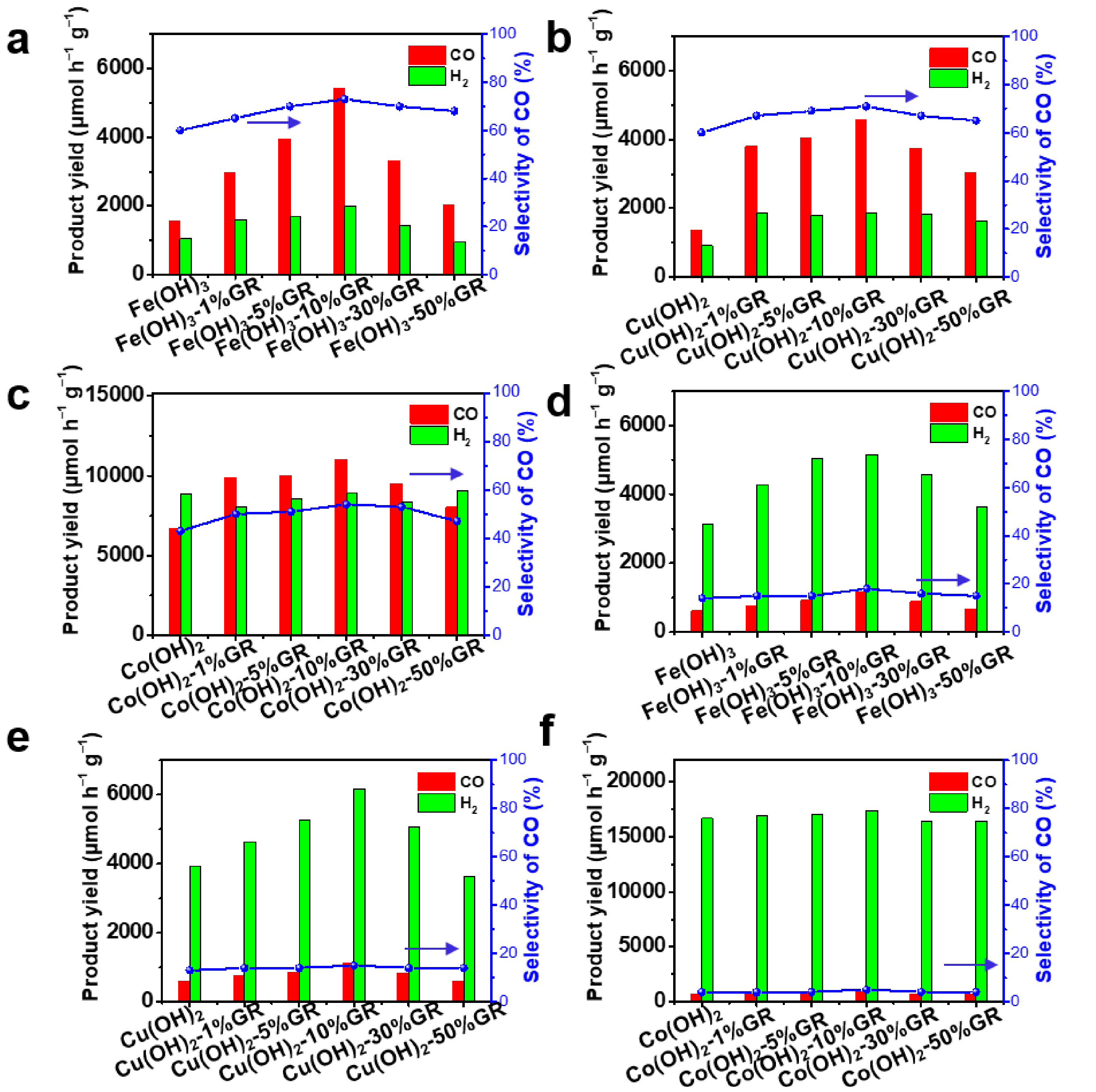

Supplementary Figure 18. CO_2 photoreduction performance of as-obtained samples CO_2 photoreduction performance over (a) $Fe(OH)_3$-GR, (b) $Cu(OH)_2$-GR and (c) $Co(OH)_2$-GR with different contents of GR in pure CO_2. CO_2 photoreduction performance over (d) $Fe(OH)_3$-GR, (e) $Cu(OH)_2$-GR and (f) $Co(OH)_2$-GR with different contents of GR in diluted CO_2.

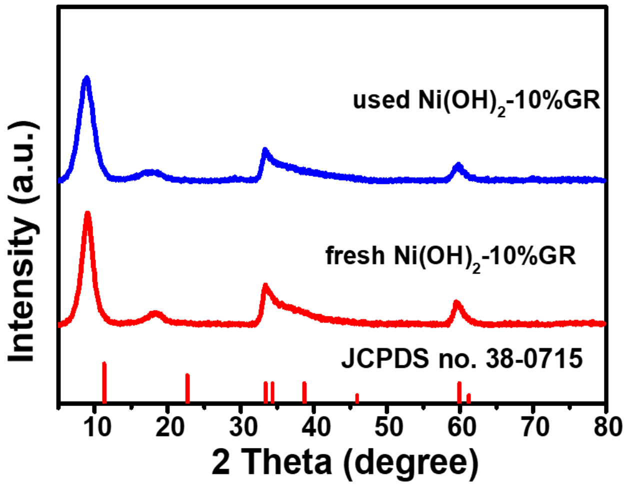

Supplementary Figure 19. XRD patterns of fresh and used $Ni(OH)_2$-10%GR composite.

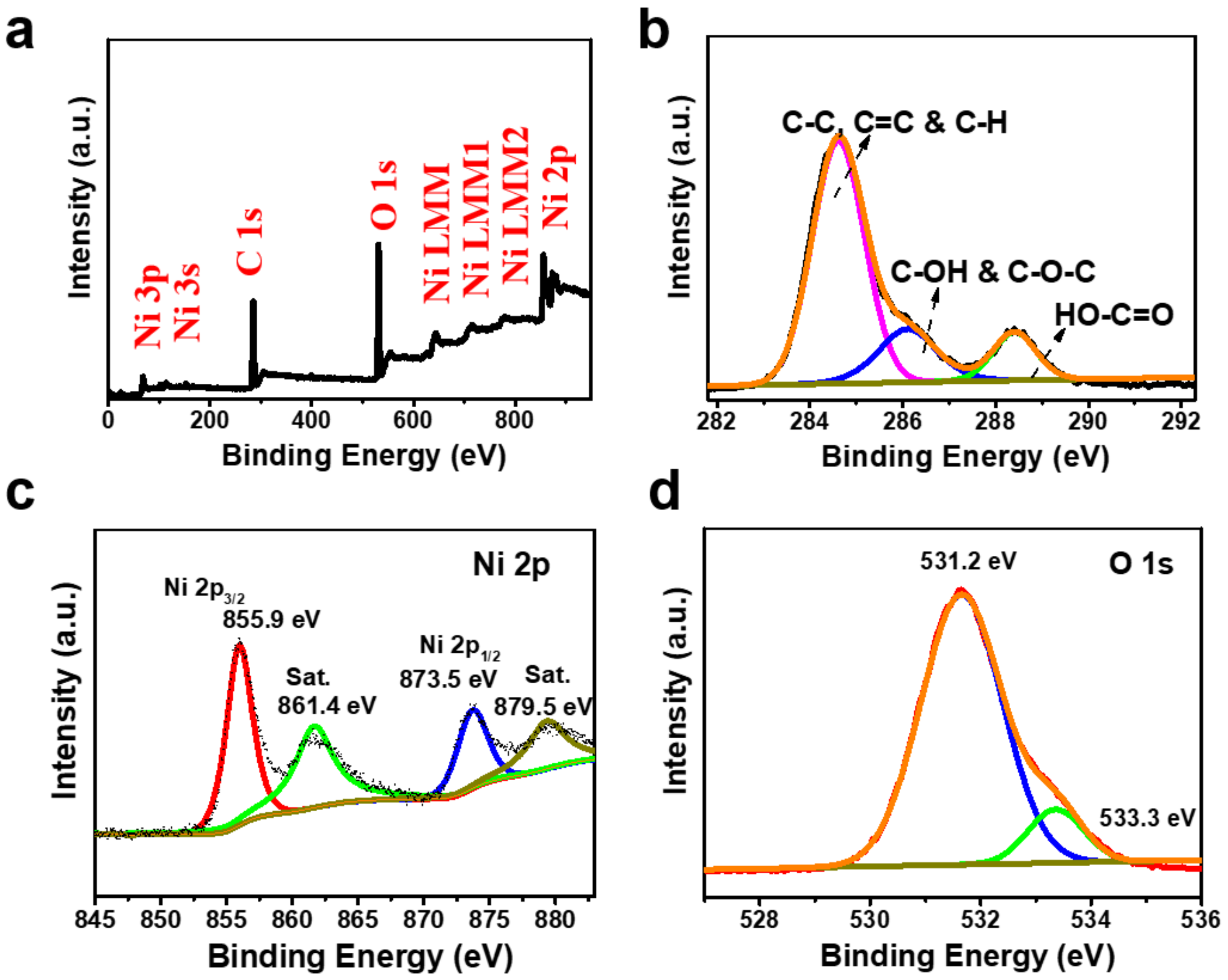

Supplementary Figure 20. XPS characterization of used $Ni(OH)_2$-10%GR composite (a) XPS survey spectrum, high-resolution XPS spectra of (b) C 1s, (c) Ni 2p and (d) O 1s of the used $Ni(OH)_2$-10%GR composite.

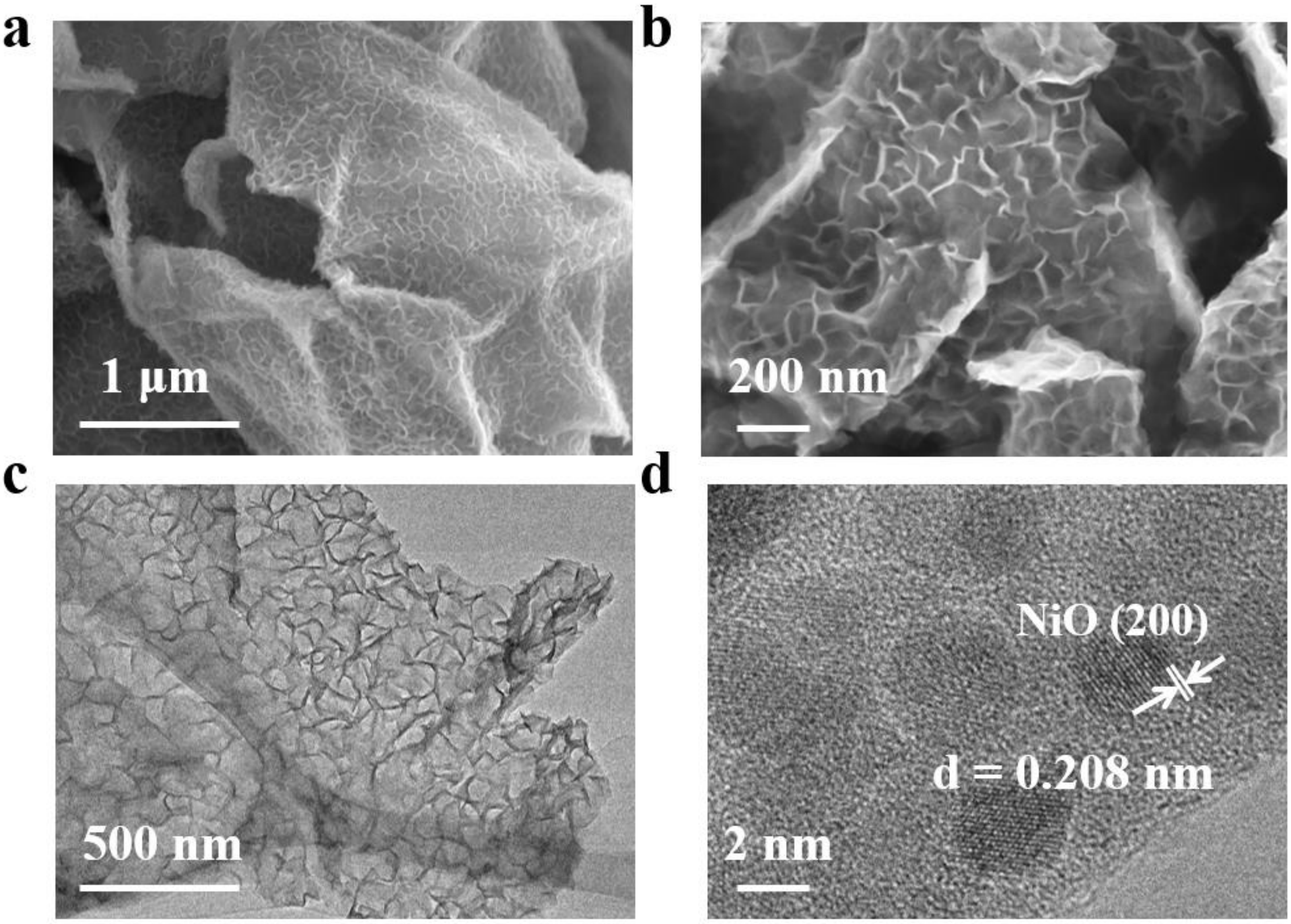

Supplementary Figure 21. Morphology information of used $Ni(OH)_2$-10%GR composite (a, b) SEM images and (c, d) TEM images of used $Ni(OH)_2$-10%GR composite.

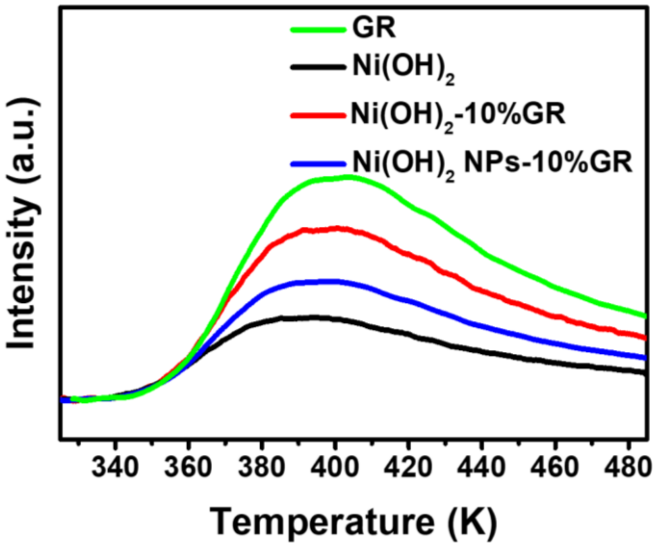

Supplementary Figure 22. CO_2 TPD test based on GR, $Ni(OH)_2$, $Ni(OH)_2$ NPs-10%GR and $Ni(OH)_2$-10%GR composite.

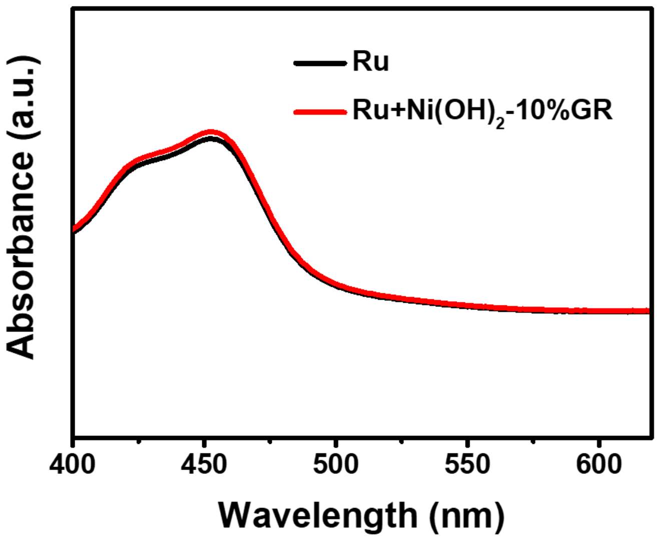

Supplementary Figure 23. UV-vis absorption spectra of Ru solution and aqueous solution containing Ru and $Ni(OH)_2$-10%GR.

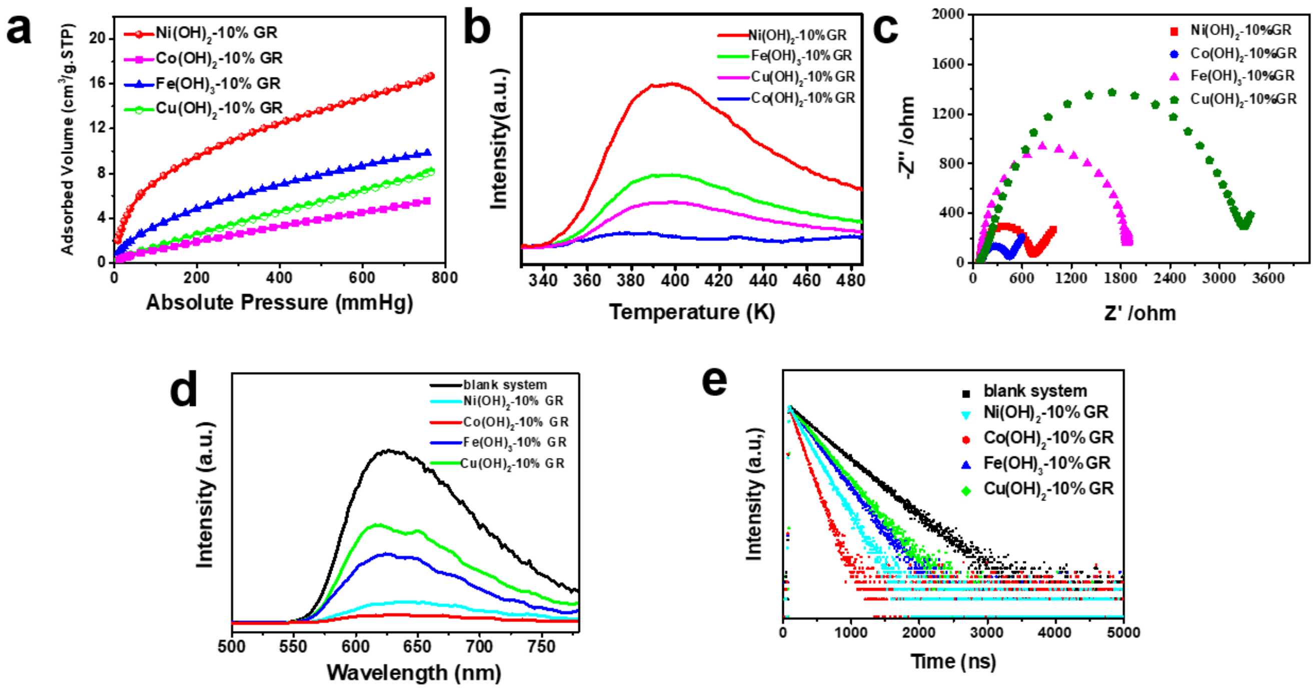

Supplementary Figure 24. CO_2 adsorption and photoelectrical properties (a) CO_2 adsorption isotherm over $Ni(OH)_2$-10%GR, $Co(OH)_2$-10%GR, $Fe(OH)_3$-10%GR and $Cu(OH)_2$-10%GR. (b) CO_2 TPD test based on $Ni(OH)_2$-10%GR, $Co(OH)_2$-10%GR, $Fe(OH)_3$-10%GR and $Cu(OH)_2$-10%GR. (c) EIS Nyquist plots of $Ni(OH)_2$-10%GR, $Co(OH)_2$-10%GR, $Fe(OH)_3$-10%GR and $Cu(OH)_2$-10%GR. (d) Steady-state PL and (e) time-resolved PL spectra decay of the photocatalytic CO_2 reduction systems with cocatalysts of $Ni(OH)_2$-10%GR, $Co(OH)_2$-10%GR, $Fe(OH)_3$-10%GR and $Cu(OH)_2$-10%GR composite and without cocatalysts.

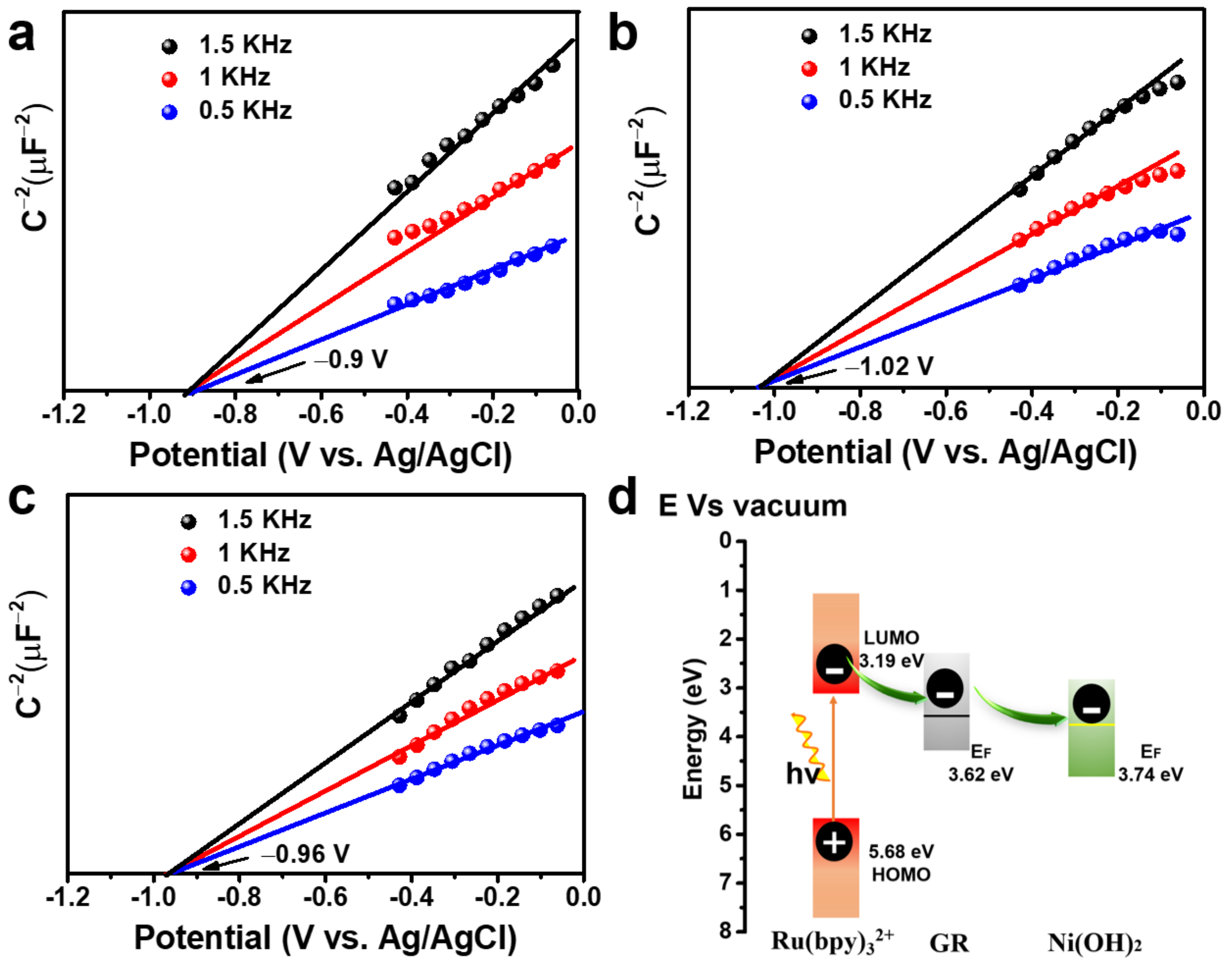

Supplementary Figure 25. Mott-Schottky plots of various photocatalysts Mott-Schottky plots of (a) $Ni(OH)_2$, (b) GR and (c) $Ni(OH)_2$-10%GR composite. (d) Schematic energy-level diagram showing the electron transfer from $[Ru(bpy)_3]Cl_2$ to GR and $Ni(OH)_2$. E_F: Fermi level; LUMO: lowest unoccupied molecular orbital; HOMO: highest occupied molecular orbital.

Supplementary Tables

Supplementary Table 1. Comparison of the performance of photocatalytic CO_2 conversion in pure CO_2.

Catalyst	Photosensitizer sacrificial agent	Reaction solvent	CO generation rate	Selectivity of CO (%)	Irradiation condition	Ref. (year)
$Ni(OH)_2$-10%GR	$[Ru(bpy)_3]Cl_2$ TEOA	CH_3CN/ H_2O	10725 μmol h^{-1} g^{-1}	96	λ ≥ 420 nm (Xe lamp)	This work
[a]CoO-Mo8	$[Ru(bpy)_3]Cl_2$ TEOA	CH_3CN/ H_2O	4165 μmol h^{-1} g^{-1}	27	λ ≥ 400 nm (Xe lamp)	[1]2020
$FeCoS_2$-CoS_2	$[Ru(bpy)_3]Cl_2$ TEOA	CH_3CN/ H_2O	28.1 μmol h^{-1}	64	λ ≥ 400 nm (Xe lamp)	[2]2020
[b]Ni-TpBpy	$[Ru(bpy)_3]Cl_2$ TEOA	CH_3CN/ H_2O	966 μmol h^{-1} g^{-1}	96	λ ≥ 420 nm (Xe lamp)	[3]2019
m-NiAl-LDH	$[Ru(bpy)_3]Cl_2$ TEOA	CH_3CN/ H_2O	712 μmol h^{-1} g^{-1}	70	λ > 400 nm (Xe lamp)	[4]2019
MOF-Ni	$[Ru(bpy)_3]Cl_2$ [c]TIPA	CH_3CN/ H_2O	317 μmol h^{-1} g^{-1}	97	λ > 400 nm (Xe lamp)	[5]2019
COF-367-Co NSs	$Ru(bpy)_3]Cl_2$ [d]AA	CH_3CN	10162 μmol h^{-1} g^{-1}	78	λ ≥ 420 nm (Xe lamp)	[6]2019
MOF-Co	$[Ru(bpy)_3]Cl_2$ TIPA	CH_3CN/ H_2O	1140 μmol h^{-1} g^{-1}	47	λ > 400 nm (Xe lamp)	[5]2019
[e]BIF-29	$[Ru(bpy)_3]Cl_2$ TEOA	CH_3CN/ H_2O	3334 μmol h^{-1} g^{-1}	83	λ > 420 nm (Xe lamp)	[7]2019
P-g-C_3N_4	–	H_2O	2.37 μmol h^{-1} g^{-1}	–	λ > 420 nm (Xe lamp)	[8]2018
[f]NC@$NiCo_2O_4$	$[Ru(bpy)_3]Cl_2$ TEOA	CH_3CN/ H_2O	26.2 μmol h^{-1}	89	λ > 420 nm (Xe lamp)	[9]2018
Ni-MOF monolayers	$[Ru(bpy)_3]Cl_2$ TEOA	CH_3CN/ H_2O	12.5 μmol h^{-1}	96	LED light	[10]2018
$CoSn(OH)_6$	$[Ru(bpy)_3]Cl_2$ TEOA	CH_3CN/ H_2O	18.7 μmol h^{-1}	58	λ ≥ 420 nm (Xe lamp)	[11]2018
[g]Ni(TPA/TEG)	$[Ru(bpy)_3]Cl_2$ TEOA	CH_3CN/ H_2O	16000 μmol h^{-1} g^{-1}	100	λ ≥ 420 nm (Xe lamp)	[12]2017
Co_3O_4	$[Ru(bpy)_3]Cl_2$ TEOA	CH_3CN/ H_2O	2003 μmol h^{-1} g^{-1}	77	λ ≥ 420 nm (Xe lamp)	[13]2016

[a] Mo8 refers to $[(nC_4H_9)N]_4Mo_8O_{26}$; [b] TpBpy refers to 2,2'-bipyridine-based covalent organic frameworks; [c] TIPA refers to triisopropanolamine; [d] AA refers to ascorbic acid; [e] BIF-29 refers to copper-based boron imidazolate cage; [f] NC refers to N-doped carbon; [g] TPA and TEG refer to terephthalic acid and triethylene glycol, respectively.

Supplementary Table 2. Comparison of the performance of photocatalytic CO_2 conversion in diluted CO_2.

Catalyst	Photosensitizer sacrificial agent	Reaction solvent	CO generation rate	Selectivity of CO (%)	Irradiation condition	Ref. (year)
$Ni(OH)_2$-10%GR	$[Ru(bpy)_3]Cl_2$ TEOA	CH_3CN/H_2O	7432 $\mu mol\ h^{-1}\ g^{-1}$	92	$\lambda \geq 420$ nm (Xe lamp)	This work
Ni-TpBpy	$[Ru(bpy)_3]Cl_2$ TEOA	CH_3CN/H_2O	288 $\mu mol\ h^{-1}\ g^{-1}$	76	$\lambda \geq 420$ nm (Xe lamp)	[3]2019
COF-367-Co NSs	$Ru(bpy)_3]Cl_2$ AA	CH_3CN/H_2O	2587 $\mu mol\ h^{-1}\ g^{-1}$	72	$\lambda \geq 420$ nm (Xe lamp)	[6]2019
Ni-MOF monolayers	$[Ru(bpy)_3]Cl_2$ TEOA	CH_3CN/H_2O	7.23 $\mu mol\ h^{-1}$	96	$\lambda \geq 420$ nm LED light	[10]2019
[a]Zr-bpdc/RuCO	$[Ru(bpy)_3](PF6)_2$ TEOA	CH_3CN	180 $\mu mol\ h^{-1}\ g^{-1}$	–	385 nm $< \lambda <$ 740 nm (Xe lamp)	[14]2016

[a] bpdc refers to biphenyl-4,4’-dicarboxylate.

Supplementary Table 3. BET surface area of GR, $Ni(OH)_2$, $Ni(OH)_2$ NPs-10%GR and $Ni(OH)_2$-10%GR.

Samples	BET surface area ($m^2\ g^{-1}$)
GR	295
$Ni(OH)_2$	11
$Ni(OH)_2$ NPs-10%GR	40
$Ni(OH)_2$-10%GR	83

Supplementary Table 4. Kinetic analysis of emission decay for blank system, GR, $Ni(OH)_2$, $Ni(OH)_2$ NPs-10%GR and $Ni(OH)_2$-10%GR.

Samples	τ_1/ns	A_1/%	τ_2/ns	A_2/%	χ^2	$\tau_{average}$/ns[a]
blank system	229	5.06	439.3	94.14	1.02	432.5
$Ni(OH)_2$	135	2.01	303	97.99	0.95	301.4
$Ni(OH)_2$ NPs -10%GR	261	90.87	389.7	9.13	1.15	260.3
$Ni(OH)_2$-10%GR	157	16.31	216	83.69	0.97	208.6
GR	91	18.79	122	81.21	1.00	117.8

[a] The average lifetime was calculated by using the equation: $\tau_{average} = (A_1\tau_1^2 + A_2\tau_2^2)/(A_1\tau_1 + A_2\tau_2)$.

Supplementary Table 5. Kinetic analysis of emission decay for $Ni(OH)_2$-10%GR, $Co(OH)_2$-10%GR, $Fe(OH)_3$-10%GR and $Cu(OH)_2$-10%GR.

Samples	τ_1/ns	A_1/%	τ_2/ns	A_2/%	χ^2	$\tau_{average}$/ns[a]
$Cu(OH)_2$-10%GR	269	68.01	339	31.45	0.94	294.8
$Fe(OH)_3$-10%GR	188	8.01	293	91.99	1.03	287.4
$Ni(OH)_2$-10%GR	157	16.31	216	83.69	0.97	208.6
$Co(OH)_2$-10%GR	89	16.79	133	83.21	1.00	127.7

[a] The average lifetime was calculated by using the equation: $\tau_{average} = (A_1\tau_1^2 + A_2\tau_2^2)/(A_1\tau_1 + A_2\tau_2)$.

Supplementary Notes

Supplementary Note 1: The dispersion of GO in water shows a strong negatively charged surface with a zeta potential value of −33.6 mV, which is stemmed from the large amount of oxygenated functional groups (*e.g.*, epoxy, carboxyl and hydroxyl groups) on the GO surface.[15] The negatively charged surface endows GO to be well dispersed in the aqueous solution and facilitates the adsorption of positively charged transition metal cations through strong electrostatic interaction between them.

Supplementary Note 2: During the synthesis process, hexamethylenetetramine (HMTA) as a hydrolyzing agent can tardily decompose to liberate OH^- ions and the reaction is described as follows[16]: $(CH_2)_6N_4 + 6\ H_2O \leftrightarrow 6\ HCHO + 4\ NH_3$, $NH_3 + H_2O \leftrightarrow NH_4^+ + OH^-$. The pH value of the initial HMTA solution is 8.51, and after 10 h of 90 ℃ reaction, the pH value of HMTA solution becomes 10.31, which clearly indicates that HMTA can decompose at this temperature.

Supplementary Note 3: As shown in **Supplementary Figure 6**, the lattice distance is measured to be 0.208 nm, corresponding to the (200) plane of NiO.[17]

Supplementary Note 4: As shown in **Supplementary Figure 10**, the weight loss below 300 °C observed for both blank $Ni(OH)_2$ and $Ni(OH)_2$-10%GR composite should be ascribed to the desorption of water on the surfaces of samples.[18] In the temperature range of 300~330 °C, an obvious weight loss is detected for both blank $Ni(OH)_2$ and $Ni(OH)_2$-10%GR composite, which can be assigned to the decomposition of $Ni(OH)_2$.[19] In addition, in the temperature range of 330~600 °C, the weight loss of $Ni(OH)_2$-10%GR composite can be assigned to the combustion of GR.[20] The content of GR in the composite can be easily calculated from the comparison of the TG results of blank $Ni(OH)_2$ and $Ni(OH)_2$-10%GR composite. It can be seen that GR content in $Ni(OH)_2$-10%GR composite is determined to be *ca.* 10.3%, which almost equals to the feedstock proportion.

Supplementary Note 5: No liquid products, such as CH_3OH, HCHO and HCOOH, are detected through analyzing the ^{1}H-NMR spectrum of the solution after photocatalytic reaction.[3]

Supplementary Note 6: As shown in **Supplementary Fig. 12**, when the content of GR is 10%, $Ni(OH)_2$-10%GR composite shows the optimal photocatalytic performance for CO_2 reduction. The lower (1% and 5%) or higher (30% and 50%) contents of GR in $Ni(OH)_2$-GR composites both result in a decreased photocatalytic performance. This could be ascribed to the fact that $Ni(OH)_2$-GR composites with lower ratio of GR display a stacked structure, which will detrimentally shield the surface active sites and augment the charge transfer resistance. As for the $Ni(OH)_2$-GR composites with higher GR content, the lower ratio of active $Ni(OH)_2$ in the hybrids still leads to the decreased catalytic performance. Notably, compared to $Ni(OH)_2$-GR composites or blank $Ni(OH)_2$, bare GR shows an inferior photocatalytic activity and CO selectivity, which indicates that main active sites for CO_2 reduction are provided by $Ni(OH)_2$ in the composites.[21]

Supplementary Note 7: As shown in **Supplementary Fig. 13**, it can be seen that $Ni(OH)_2$ NPs-10%GR composite shows well-resolved diffraction peaks of α-$Ni(OH)_2$ (JCPDS no. 38-0715).[22,23]

Supplementary Note 8: As shown in **Supplementary Fig. 14**, $Ni(OH)_2$ NPs-10%GR composite shows similar XPS spectra as compared to that of $Ni(OH)_2$-10%GR composite. Notably, the C 1s XPS spectrum indicates effective reduction of GR in $Ni(OH)_2$ NPs-10%GR composite, manifesting the different photocatalytic performance between $Ni(OH)_2$-10%GR and $Ni(OH)_2$ NPs-10%GR composite is not due to the different reduction degree of GR.

Supplementary Note 9: As shown in **Supplementary Fig. 15**, the SEM images of $Ni(OH)_2$ NPs-10%GR composite show that $Ni(OH)_2$ NPs-10%GR composite is composed of aggregated $Ni(OH)_2$ nanoparticles grown on the GR surface.

Supplementary Note 10: Supplementary Fig. 16 shows the FESEM images of the $Fe(OH)_3$-10%GR, $Cu(OH)_2$-10%GR and $Co(OH)_2$-10%GR composites. It can be seen that $Fe(OH)_3$ and $Cu(OH)_2$ grow on GR in the form of irregular nanosheet, while $Co(OH)_2$ grows on GR with the morphology of nanosheet array. The different morphology of these transition metal hydroxides may be due to their different solubility product constant (K_{sp}). The K_{sp} of $Fe(OH)_3$ and $Cu(OH)_2$ are relatively small with 4×10^{-33} and 2.2×10^{-20} respectively, which causes them to rapidly nucleate and grow into irregular nanosheet, while the larger K_{sp} of $Ni(OH)_2$ (2×10^{-15}) and $Co(OH)_2$ (5.9×10^{-15}) enables them to slowly nucleate and grow into nanosheet arrays.[24,25]

Supplementary Note 11: XRD patterns in **Supplementary Fig. 17** suggest that $Fe(OH)_3$-GR, $Cu(OH)_2$-GR and $Co(OH)_2$-GR show well-resolved diffraction peaks of $Fe(OH)_3$, $Cu(OH)_2$ and $Co(OH)_2$ and the diffraction peaks of GR are not observed in these composites, indicating that GR layers are densely wrapped by $Fe(OH)_3$, $Cu(OH)_2$ and $Co(OH)_2$.

Supplementary Note 12: As shown in **Supplementary Fig. 25a-c**, Mott-Schottky plots measured at frequency of 0.5 KHz, 1 KHz and 1.5 KHz indicate that the flat band potential of $Ni(OH)_2$, GR and $Ni(OH)_2$-10%GR is calculated to be *ca.* −0.9 V, −1.02 V and −0.96 V *vs.* Ag/AgCl, corresponding to −0.7 V, −0.82 V and −0.76 V *vs.* normal hydrogen electrode (NHE).[11,26] According to the relationship between the normal electrode potential and the absolute vacuum energy with the numerical difference of 4.44,[27] flat band potential of $Ni(OH)_2$, GR and $Ni(OH)_2$-10%GR is calculated to be 3.74, 3.62 and 3.68 eV *vs.* vacuum level. In addition, because the obtained flat-band potentials is equal to the fermi level (E_F),[21,28,29] the E_F values of $Ni(OH)_2$ and GR are *ca.* 3.74 and 3.62 eV respectively (*vs.* vacuum level). Furthermore, the energy levels of $[Ru(bpy)_3]Cl_2$ with respect to the vacuum level have been confirmed as 3.19 eV for the lowest unoccupied molecular orbital (LUMO) and 5.68 eV for the highest occupied molecular orbital (HOMO) in previous researches.[27,30] Based on the above results, the energy level diagram and the electron transfer mechanism are illustrated in **Supplementary Fig. 25d**.

Supplementary References:

1. Wang, X., Yang, H. & Yang, D. POM-incorporated CoO nanowires for enhanced photocatalytic syngas production from CO_2. *Angew. Chem. Int. Ed.* (2020) 10.1002/anie.202004563.
2. Wang, Y., Wang, S., Zhang, S.L. et al. Formation of hierarchical $FeCoS_2$-CoS_2 double-shelled nanotubes with enhanced performance for photocatalytic reduction of CO_2. *Angew. Chem. Int. Ed. Engl.* **59**, 11918-11922 (2020).
3. Zhong, W., Sa, R., Li, L. et al. A covalent organic framework bearing single Ni sites as a synergistic photocatalyst for selective photoreduction of CO_2 to CO. *J. Am. Chem. Soc.* **141**, 7615-7621 (2019).
4. Tan, L., Xu, S.M., Wang, Z. et al. Highly selective photoreduction of CO_2 with suppressing H_2 evolution over monolayer layered double hydroxide under irradiation above 600 nm. *Angew. Chem. Int. Ed.* **131**, 11986-11993 (2019).
5. Wang, X.-K., Liu, J., Zhang, L. et al. Monometallic catalytic models hosted in stable metal-organic frameworks for tunable CO_2 photoreduction. *ACS Catal.* **9**, 1726-1732 (2019).
6. Liu, W., Li, X., Wang, C. et al. A scalable general synthetic approach toward ultrathin imine-linked two-dimensional covalent organic framework nanosheets for photocatalytic CO_2 reduction. *J. Am. Chem. Soc.* **141**, 17431-17440 (2019).
7. Zhang, H.-X., Hong, Q.-L., Li, J. et al. Isolated square-planar copper center in boron imidazolate nanocages for photocatalytic reduction of CO_2 to CO. *Angew. Chem. Int. Ed.* **131**, 11878-11882 (2019).
8. Liu, B., Ye, L., Wang, R. et al. Phosphorus-doped graphitic carbon nitride nanotubes with amino-rich surface for efficient CO_2 capture, enhanced photocatalytic activity, and product selectivity. *ACS Appl. Mater. Interfaces* **10**, 4001-4009 (2018).
9. Wang, S., Guan, B.Y. & Lou, X.W. Rationally designed hierarchical N-doped carbon@$NiCo_2O_4$ double-shelled nanoboxes for enhanced visible light CO_2 reduction. *Energy Environ. Sci.* **11**, 306-310 (2018).
10. Han, B., Ou, X., Deng, Z. et al. Nickel metal-organic framework monolayers for photoreduction of diluted CO_2: metal-node-dependent activity and selectivity. *Angew. Chem. Int. Ed.* **57**, 16811-16815 (2018).
11. Lin, X., Gao, Y., Jiang, M. et al. Photocatalytic CO_2 reduction promoted by uniform perovskite hydroxide $CoSn(OH)_6$ nanocubes. *Appl. Catal., B* **224**, 1009-1016 (2018).
12. Niu, K., Xu, Y., Wang, H. et al. A spongy nickel-organic CO_2 reduction photocatalyst for nearly 100% selective CO production. *Sci. Adv.* **3**, e1700921 (2017).
13. Gao, C., Meng, Q., Zhao, K. et al. Co_3O_4 hexagonal platelets with controllable facets enabling highly efficient visible-light photocatalytic reduction of CO_2. *Adv. Mater.* **28**, 6485-6490 (2016).
14. Kajiwara, T., Fujii, M., Tsujimoto, M. et al. Photochemical reduction of low concentrations of CO_2 in a porous coordination polymer with a ruthenium(ii)-CO complex. *Angew. Chem. Int. Ed.* **55**, 2697-2700 (2016).
15. Zhang, N., Yang, M.-Q., Liu, S. et al. Waltzing with the versatile platform of graphene to synthesize composite photocatalysts. *Chem. Rev.* **115**, 10307-10377 (2015).
16. Nagaraju, G., Cha, S.M. & Yu, J.S. Ultrathin nickel hydroxide nanosheet arrays grafted biomass-derived honeycomb-like porous carbon with improved electrochemical performance as a supercapacitive material. *Sci. Rep.* **7**, 45201 (2017).
17. Hussain, N., Yang, W., Dou, J. et al. Ultrathin mesoporous F-doped α-$Ni(OH)_2$ nanosheets as an

efficient electrode material for water splitting and supercapacitors. *J. Mater. Chem. A* **7**, 9656-9664 (2019).

18. Weng, B., Wu, J., Zhang, N. et al. Observing the role of graphene in boosting the two-electron reduction of oxygen in graphene-WO_3 nanorod photocatalysts. *Langmuir* **30**, 5574-5584 (2014).
19. Liu, Y., Wang, R. & Yan, X. Synergistic effect between ultra-small nickel hydroxide nanoparticles and reduced graphene oxide sheets for the application in high-performance asymmetric supercapacitor. *Sci. Rep.* **5**, 11095 (2015).
20. Wang, R., Jayakumar, A., Xu, C. et al. $Ni(OH)_2$ nanoflowers/graphene hydrogels: a new assembly for supercapacitors. *ACS Sustain. Chem. Eng.* **4**, 3736-3742 (2016).
21. Mu, Q., Zhu, W., Li, X. et al. Electrostatic charge transfer for boosting the photocatalytic CO_2 reduction on metal centers of 2D MOF/rGO heterostructure. *Appl. Catal., B* **262**, 118144 (2020).
22. Jia, D., Gao, H., Dong, W. et al. Hierarchical α-$Ni(OH)_2$ composed of ultrathin nanosheets with controlled interlayer distances and their enhanced catalytic performance. *ACS Appl. Mater. Interfaces* **9**, 20476-20483 (2017).
23. Tang, T., Jiang, W.-J., Niu, S. et al. Kinetically controlled coprecipitation for general fast synthesis of sandwiched metal hydroxide nanosheets/graphene composites toward efficient water splitting. *Adv. Funct. Mater.* **28**, 1704594 (2018).
24. Li, Y., Wu, T., Jiang, K. et al. Mn^{2+} induced structure evolution and dual-frequency microwave absorption of $Mn_xFe_{3-x}O_4$ hollow/porous spherical chains made by a one-pot solvothermal approach. *J. Mater. Chem. C* **4**, 7119-7129 (2016).
25. Xie, M., Duan, S., Shen, Y. et al. In-situ-grown $Mg(OH)_2$-derived hybrid α-$Ni(OH)_2$ for highly stable supercapacitor. *ACS Energy Lett.* **1**, 814-819 (2016).
26. Han, B., Song, J., Liang, S. et al. Hierarchical $NiCo_2O_4$ hollow nanocages for photoreduction of diluted CO_2: Adsorption and active sites engineering. *Appl. Catal., B* **260**, 118208 (2020).
27. Chen, W., Han, B., Xie, Y. et al. Ultrathin Co-Co LDHs nanosheets assembled vertically on MXene: 3D nanoarrays for boosted visible-light-driven CO_2 reduction. *Chem. Eng. J.*, 123519 (2019).
28. Xie, Y., Fang, Z., Li, L. et al. Creating chemisorption sites for enhanced CO_2 photoreduction activity through alkylamine modification of MIL-101-Cr. *ACS Appl. Mater. Interfaces* **11**, 27017-27023 (2019).
29. Zhang, W., Li, W., Li, Y. et al. One-step synthesis of nickel oxide/nickel carbide/graphene composite for efficient dye-sensitized photocatalytic H_2 evolution. *Catal. Today* **335**, 326-332 (2019).
30. Gao, C., Chen, S., Wang, Y. et al. Heterogeneous single-atom catalyst for visible-light-driven high-turnover CO_2 reduction: the role of electron transfer. *Adv. Mater.* **30**, 1704624 (2018).
